# Uric Acid Predicts Recovery of Left Ventricular Function and Adverse Events in Heart Failure With Reduced Ejection Fraction: Potential Mechanistic Insight From Network Analyses

**DOI:** 10.3389/fcvm.2022.853870

**Published:** 2022-07-15

**Authors:** Xiqiang Wang, Xiude Fan, Qihui Wu, Jing Liu, Linyan Wei, Dandan Yang, Xiang Bu, Xiaoxiang Liu, Aiqun Ma, Tomohiro Hayashi, Gongchang Guan, Yu Xiang, Shuang Shi, Junkui Wang, Jiansong Fang

**Affiliations:** ^1^Department of Cardiovascular Medicine, Shaanxi Provincial People's Hospital, Xi'an, China; ^2^Department of Endocrinology, Shandong Provincial Hospital Affiliated to Shandong First Medical University, Jinan, China; ^3^Shandong Clinical Research Center of Diabetes and Metabolic Diseases, Jinan, China; ^4^Shandong Key Laboratory of Endocrinology and Lipid Metabolism, Jinan, China; ^5^Science and Technology Innovation Center, Guangzhou University of Chinese Medicine, Guangzhou, China; ^6^Clinical Research Center, Hainan Provincial Hospital of Traditional Chinese Medicine, Guangzhou University of Chinese Medicine, Haikou, China; ^7^Department of Cardiovascular Medicine, First Affiliated Hospital of Xi'an Jiaotong University, Xi'an, China; ^8^Department of General Practice, The Second Affiliated Hospital Zhejiang University School of Medicine, Hangzhou, China; ^9^Department of Cardiovascular Medicine, The Second Affiliated Hospital of Zhejiang University, Hangzhou, China; ^10^Center for Cardiovascular Research, Cardiovascular Division, Department of Medicine, Washington University School of Medicine, St. Louis, MO, United States

**Keywords:** uric acid, network analyses, heart failure with a reduced ejection fraction, recovery of left ventricular ejection fraction, heart failure with recovery ejection fraction

## Abstract

**Background and Aims:**

Heart failure with reduced ejection fraction (HFrEF) still carries a high risk for a sustained decrease in left ventricular ejection fraction (LVEF) even with the optimal medical therapy. Currently, there is no effective tool to stratify these patients according to their recovery potential. We tested the hypothesis that uric acid (UA) could predict recovery of LVEF and prognosis of HFrEF patients and attempted to explore mechanistic relationship between hyperuricemia and HFrEF.

**Methods:**

HFrEF patients with hyperuricemia were selected from the National Inpatient Sample (NIS) 2016–2018 database and our Xianyang prospective cohort study. Demographics, cardiac risk factors, and cardiovascular events were identified. Network-based analysis was utilized to examine the relationship between recovery of LVEF and hyperuricemia, and we further elucidated the underlying mechanisms for the impact of hyperuricemia on HFrEF.

**Results:**

After adjusting confounding factors by propensity score matching, hyperuricemia was a determinant of HFrEF [OR 1.247 (1.172–1.328); *P* < 0.001] of NIS dataset. In Xianyang prospective cohort study, hyperuricemia is a significant and independent risk factor for all-cause death (adjusted HR 2.387, 95% CI 1.141–4.993; *P* = 0.021), heart failure readmission (adjusted HR 1.848, 95% CI 1.048–3.259; *P* = 0.034), and composite events (adjusted HR 1.706, 95% CI 1.001–2.906; *P* = 0.049) in HFrEF patients. UA value at baseline was negatively correlated to LVEF of follow-ups (*r* = −0.19; *P* = 0.046). Cutoff UA value of 312.5 μmmol/L at baseline can work as a predictor of LVEF recovery during follow-up, with the sensitivity of 66.7%, the specificity of 35.1%, and the accuracy of 0.668 (95% CI, 0.561–0.775; *P* = 0.006). Moreover, gene overlap analysis and network proximity analysis demonstrated a strong correlation between HFrEF and Hyperuricemia.

**Conclusion:**

Lower baseline UA value predicted the LVEF recovery and less long-term adverse events in HFrEF patients. Our results provide new insights into underlying mechanistic relationship between hyperuricemia and HFrEF.

## Introduction

Medical regimen can improve the clinical outcomes of heart failure with reduced ejection fraction (HFrEF) and lead to a reverse left ventricular (LV) remodeling in HFrEF patients (1, 2). However, the degree of reverse remodeling is extremely variable, some patients might not have any reverse remodeling with persistently reduced LV ejection fraction (LVEF) and heart failure phenotype, whereas other patients can have completely myocardial recovery with increased LVEF, decreased LV volume and mass (1–4). At present, clinical variables and validated clinical indices cannot accurately predict the direction or extent of LVEF recovery after treated with optimal pharmacologic therapy. Thus, the identification of potential responders to therapy vs. non-responders remains a major unmet need.

Uric acid (UA) is a metabolic decomposition product of purine nucleotides, the relationship between serum UA and cardiovascular diseases, especially for heart failure (HF), has received extensive attention during past few decades (5). Various studies have demonstrated that hyperuricemia was related with higher morbidity and mortality in chronic HF (6–8) and acute HF (9, 10). It was previously proposed that the higher UA in chronic HF is resulted from the up-regulation of the xanthine oxidase, which is an important enzyme for UA metabolism and oxygen free radicals production. Increased production of the oxygen free radicals can lead to impaired vascular function (11), oxidative metabolism dysfunction (12), and inflammatory cytokine activation (13). However, there is still a lack of UA cut-off value to predict LVEF recovery and long-term prognosis of HFrEF patients in clinical practice, and the mechanistic relationship between hyperuricemia and HFrEF remains unclear.

Here, we hypothesize that UA is potentially associate with the recovery of LVEF, serves as an important predictor for the LVEF recovery and prognosis in HFrEF patients. To this end, we investigated the association of hyperuricemia and HFrEF using data from the National Inpatient Sample (NIS) database and our Xianyang prospective cohort study. Additionally, we performed gene overlap and protein-protein interaction network analyses to explore the underlying mechanistic relationship between hyperuricemia and HFrEF.

## Materials and Methods

A detailed methods section, including Supplementary Tables S1, S2.

## Results

### Characteristics of Study Participants Selected From NIS Database

Hyperuricemia was present in 0.74% (*n* = 13,468) of in-hospital admissions and it was more common in patients with HFrEF than the patients without HFrEF (0.2 vs. 0.07%; *P* < 0.001). HFrEF patients were older (69.49 vs.57.29; *P* < 0.001) and less likely to be females (38.6 vs. 58.8%; *P* < 0.0001) compared with patients without HFrEF. Moreover, these HFrEF patients suffered more from most traditional cardiac risk factors, including obesity (15.3 vs.17.3%; *P* < 0.0001), hyperlipidemia (51.8 vs. 31.3%; *P* < 0.0001), tobacco use (28.8 vs.19.9%; *P* < 0.0001), peripheral vascular disease (2.9 vs.1.5%; *P* < 0.0001), carotid artery disease (1.9 vs.1.3%; *P* < 0.0001), prior myocardial infarction (19.5 vs.4.8%; *P* < 0.0001), prior coronary aorta bypass graft (CABG) (26.9 vs.7.9%; *P* < 0.0001). An increased in-hospital mortality (5.1 vs. 2.1%; *P* < 0.0001), length of hospital stay (6.37 vs. 4.66%; *P* < 0.0001) and total medical expenses (79,313.65 vs. 52,283.83; *P* < 0.0001) were observed in HFrEF patients compared to the patients without HFrEF (Supplementary Table S4).

### Hyperuricemia Is Associated With the Risk of HFrEF and In-hospital Complications in NIS Database

**Figure 1:**
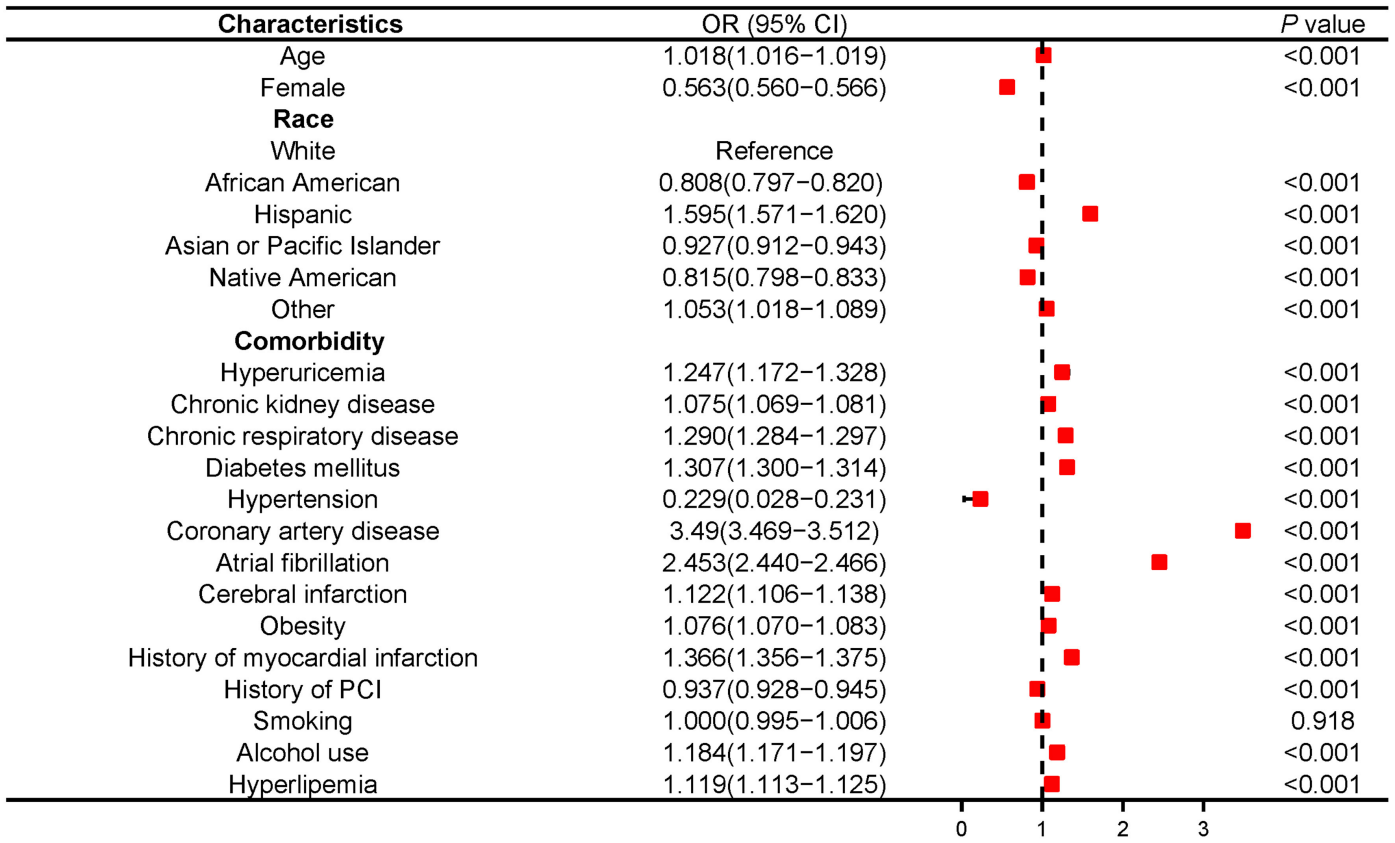
Multivariable logistic analysis results of relationship between hyperuricemia and HFrEF in NIS database 2016-2018. PCI, Percutaneous coronary intervention.

Regression analysis demonstrated that hyperuricemia was a determinant of HFrEF [OR 2.138 (2.019–2.55); *P* < 0.001] (Supplementary Figure S2), and hyperuricemia remained strongly associated with HFrEF after adjusting for other confounding risk factors, including age, gender, race, diabetes mellitus, hypertension, hyperlipidemia, obesity, tobacco use, coronary artery disease, atrial fibrillation, peripheral vascular diseases, carotid artery stenosis, cerebral infarction, myocardial infarction, stroke, prior percutaneous coronary intension (PCI) and prior CABG [OR 1.247 (1.172–1.328); *P* < 0.001] (Figure 1).

To determine whether hyperuricemia in HFrEF patients leads to higher risk of in-hospital mortality, acute organ failure and mechanical circulatory support, propensity score matching (PSM) was applied to reduce the bias due to confounding variables (Table 1). The results demonstrated that HFrEF patients with hyperuricemia presented with longer length of hospital stay (7.60 vs. 6.45; *P* < 0.0001), more acute respiratory failure (17.39 vs. 13.42%; *P* = 0.006), acute renal failure (67.59 vs. 36.70%; *P* < 0.0001), sudden cardiac arrest (1.75 vs. 0.79%; *P* = 0.033), ventilator use (7.94 vs. 3.57%; *P* < 0.0001), intra-aortic balloon pump (IABP) implantation (1.43 vs. 0.32%; *P* = 0.003), and PCI (3.65 vs. 2.14%; *P* = 0.024). However, there was no significant difference in hospital mortality between HFrEF patients with and without hyperuricemia (4.37 vs. 5.65%; *P* = 0.169) (Table 1).

**Table 1:** Demographics and outcomes of patients with Heart Failure with Reduced Ejection Fraction, National Inpatient Sample (NIS) 2016–2018.

	**Unmatched groups**			**Propensity-matched groups**		
**Characteristic**	**HFrEF without hyperuricemia (*n* = 865,772)**	**HFrEF with hyperuricemia (*n* = 1,302)**	***P-*value**	**HFrEF without hyperuricemia (*n* = 1,259)**	**HFrEF with hyperuricemia (*n* = 1,259)**	***P-*value**
Age, yrs (mean ± SD)	69.49 ± 14.16	69.12 ± 14.16	0.342	68.10 ± 14.18	69.07 ± 14.16	0.085
Female, %	318,904 (36.8)	433 (33.3)	0.007	412 (32.72)	423 (33.60)	0.641
**Race**			<0.001			0.464
White, %	562,256 (66.9)	777 (61.2)		777 (61.72)	771 (61.24)	
African American, %	164,692 (19.6)	280 (22.0)		278 (22.08)	276 (21.92)	
Hispanic, %	69,547 (8.3)	116 (9.1)		106 (8.42)	116 (9.21)	
Asian/Pacific Islander, %	17,527 (2.1)	48 (3.8)		35 (2.78)	48 (3.81)	
Native American, %	4,751 (0.6)	6 (0.5)		9 (0.71)	6 (0.48)	
Other races, %	21,905 (2.6)	43 (3.4)		54 (4.29)	42 (3.34)	
**Comorbidity**						
Coagulopathy, %	25,481 (2.9)	52 (4.0)	0.025	53 (4.21)	50 (3.97)	0.763
Obesity, %	149,757 (17.3)	338 (26)	<0.001	312 (24.78)	325 (25.81)	0.551
Hypertension, %	117,078 (13.5)	84 (6.5)	<0.001	82 (6.51)	83 (6.59)	0.936
Hypothyroidism, %	128,087 (14.8)	220 (16.9)	0.033	208 (16.52)	214 (17.00)	0.749
Coronary artery disease, %	530,813 (61.3)	775 (59.5)	0.186	721 (57.27)	749 (59.49)	0.258
Atrial fibrillation, %	374,078 (43.2)	546 (41.9)	0.355	495 (39.32)	523 (41.54)	0.256
Diabetes mellitus, %	390,624 (45.1)	624 (47.9)	0.042	612 (48.61)	606 (48.13)	0.811
Cerebral infarction, %	24,196 (2.8)	21 (1.6)	0.01			
Peripheral vascular disease, %	24,679 (2.9)	38 (2.9)	0.883	27 (2.14)	37 (2.94)	0.205
Hypercholesteremia, %	448,279 (51.8)	716 (55.0)	0.02	692 (54.96)	685 (54.41)	0.779
Alcohol use, %	40,648 (4.7)	50 (3.8)	0.145	50 (3.97)	48 (3.81)	0.837
Tobacco abuse, %	249,362 (28.8)	387 (29.7)	0.463	358 (28.44)	371 (29.47)	0.568
In-hospital mortality, %	44,085 (5.1)	56 (4.3)	0.195	70 (5.56)	55 (4.37)	0.169
Length of hospital stay, days	6.37 ± 7.72	7.61 ± 6.79	<0.001	6.45 ± 7.27	7.60 ± 6.74	<0.001
Total charges, US$	79,306.03 ± 141,173.79	84,376.30 ± 118,821.790	0.197	87,196.87 ± 192,315.06	85,116.90 ± 119,822.18	0.745
**Acute organ failure**						
Acute respiratory failure, %	138,930 (16.0)	174 (13.4)	0.008	169 (13.42)	219 (17.39)	0.006
Acute renal failure, %	287,968 (33.3)	883 (67.8)	<0.001	462 (36.70)	851 (67.59)	<0.001
Acute hepatic failure, %	13,956 (1.6)	30 (2.3)	0.048	27 (2.14)	29 (2.30)	0.787
Acute pulmonary edema, %	4,420 (0.5)	8 (0.6)	0.599	7 (0.56)	8 (0.64)	0.796
Hematodialysis, %	58,169 (6.7)	121 (9.3)	<0.001			
Ventricular fibrillation, %	11,795 (1.4)	12 (0.9)	0.17	22 (1.75)	12 (0.95)	0.084
Cardiac shock, %	36,546 (4.2)	55 (4.2)	0.996	61 (4.85)	54 (4.29)	0.504
Sudden cardiac arrest, %	18,184 (2.1)	11 (0.8)	0.002	9.9 (0.79)	22 (1.75)	0.033
Cardio-pulmonary resuscitation, %	10,694 (1.2)	8 (0.6)	0.043	11 (0.87)	8 (0.64)	0.49
Ventilator use, %	64,044 (7.4)	46 (3.5)	<0.001	45 (3.57)	100 (7.94)	<0.001
**Mechanical circulatory support**						
LVAD, %	1,693 (0.2)	4 (0.4)	0.362	4 (0.32)	4 (0.32)	1
IABP, %	8,872 (1.0)	5 (0.4)	0.022	4 (0.32)	18 (1.43)	0.003
ECMO, %	1,344 (0.2)	0 (0)	0.155	5 (0.40)	0 (0.00)	0.062
PCI, %	39,174 (4.5)	27 (2.1)	<0.001	27 (2.14)	46 (3.65)	0.024

*CABG, coronary artery bypass grafting; LVAD, left ventricular assist device; PCI, percutaneous coronary intervention; MI, myocardial infarction; ECHO, extracorporeal membrane oxygenation*.

### Hyperuricemia Associated With the Long-Term Outcomes of HFrEF Patients in the Xianyang Chronic Heart Failure Prospective Cohort

As shown in Supplementary Figure S5, a total of 102 patients met our inclusion criteria, with 86 patients (84.3%) in UA ≤ 420 ummol/L group and 16 patients (15.7%) in UA > 420 ummol/L group. The average age of participants was 61.1 and 61.7% of them were male (Supplementary Table S5). Characteristics of the patients in this study were shown in Supplementary Table S5.

There were 102 patients followed up with a mean duration of 33.44 months. During that time, 26 patients (25.5%) died, 59 patients (57.84%) had remission. The UA ≥ 420 μmmol/L group saw increased all-cause death rate (43.8 vs. 22.1%; *P* = 0.068), HF readmission (81.3 vs. 56.1%; *P* = 0.06) and composite outcomes events (87.5 vs. 65.1%; *P* = 0.076) (Supplementary Table S5).

**Figure 2:**
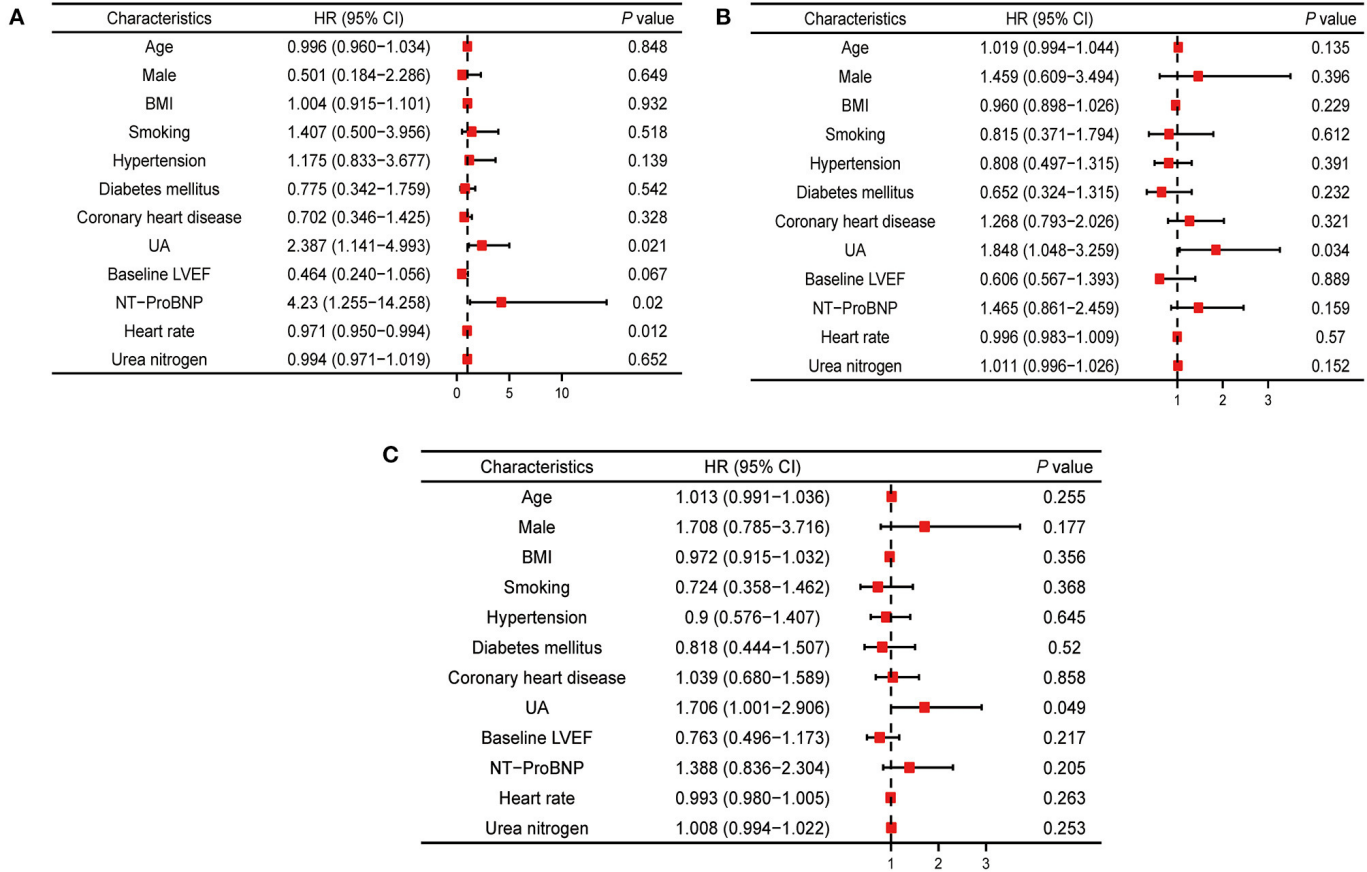
Multivariable cox analysis for hazard ratios of outcomes associated with UA. **(A)** Multivariable cox analysis results of relationship between UA and all-cause mortality. **(B)** Multivariable cox analysis results of relationship between UA and heart failure readmission. **(C)** Multivariable cox analysis results of relationship between UA and composite outcomes events. BMI, body mass index; LVEF, left ventricle ejection fraction; UA, uric acid; NT-proBNP, N-terminal pro brain natriuretic peptide.

Multivariate Cox regression analysis showed that HFrEF patients with hyperuricemia (UA ≥ 420 μmmol/L) had higher risks for all-cause death (adjusted HR 2.387, 95% CI 1.141–4.993; *P* = 0.021), HF readmission (adjusted HR 1.848, 95% CI 1.048–3.259; *P* = 0.034), and composite events (adjusted HR 1.706, 95% CI 1.001–2.906; *P* = 0.049) compared with HFrEF patients with normal serum UA (Figure 2).

**Figure 3:**
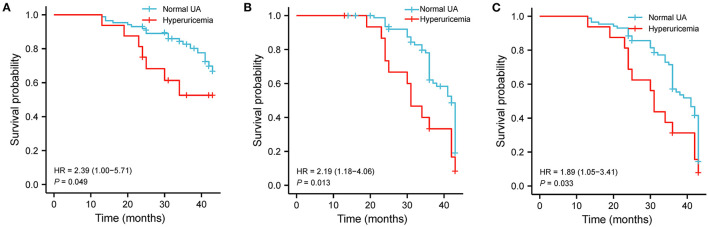
Hyperuricemia associated with clinical events in HFrEF patients. **(A)** Kaplan–Meier survival plots for the all-cause death. **(B)** Readmission due to worsening HF. **(C)** Composite end points in hyperuricemia and normal UA groups. UA, uric acid.

Kaplan-Meier survival curves displayed different event free survival for all-cause death (HR = 2.39, log-rank *P* = 0.049, Figure 3A), readmission (HF = 2.19, log-rank *P* = 0.013, Figure 3B), and composite events (HR = 1.89, log-rank *P* = 0.033, Figure 3C) between HFrEF patients with and without hyperuricemia.

### Hyperuricemia Is Associated With the Recovery of LVEF in HFrEF Patients in the Xianyang Chronic Heart Failure Prospective Cohort

**Figure 4:**
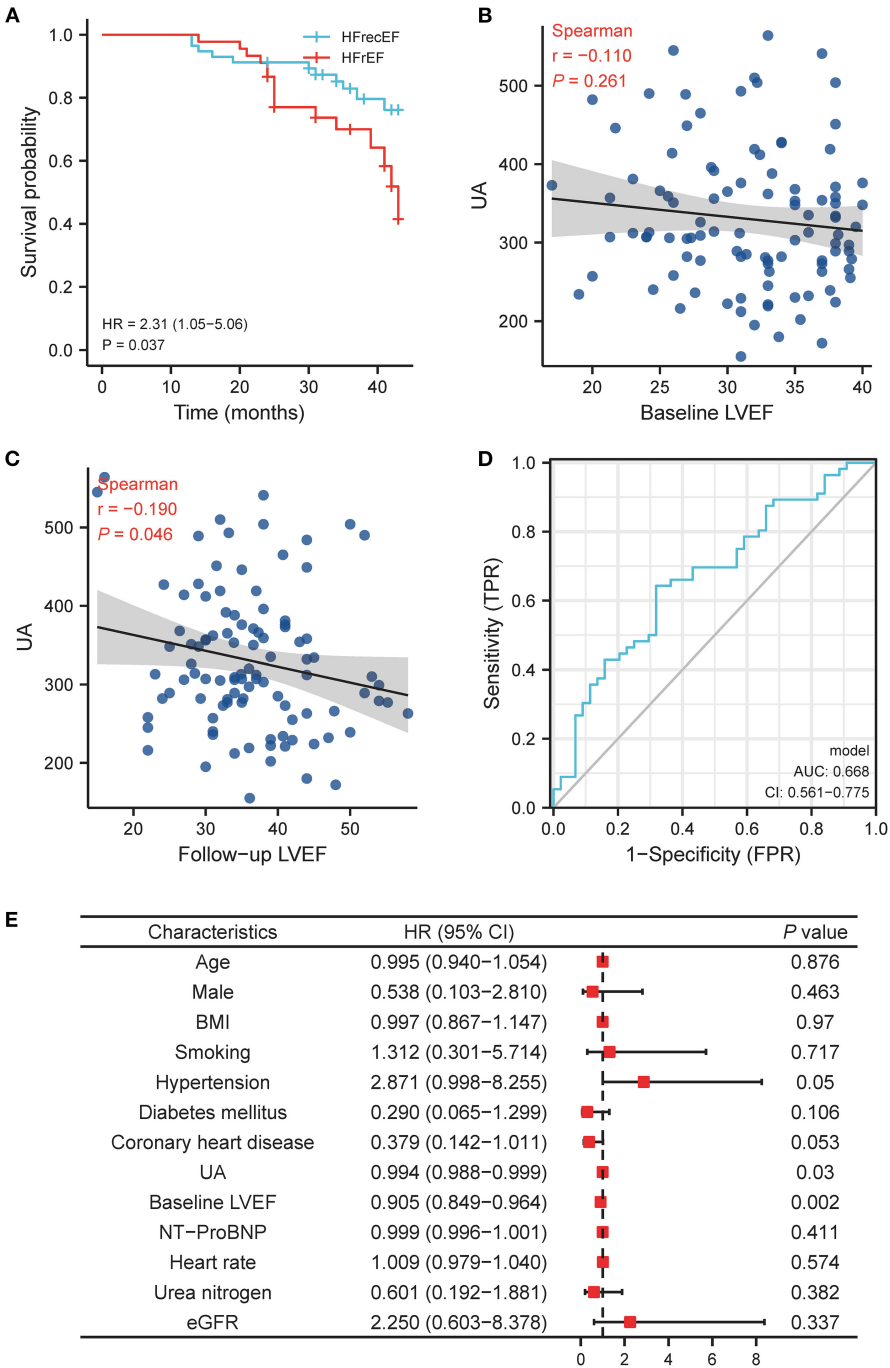
The relationship between UA and left ventricle ejection fraction recovery in HFrEF patient. **(A)** Kaplan-Meier survival plots for the all-cause death in persistently HFrEF and HFrecEF patients. **(B)** The relationship between UA and LVEF at baseline LVEF. **(C)** The relationship between UA and LVEF at follow-up LVEF. **(D)** ROC curve for baseline UA and follow-up LVEF. **(E)** Multivariable logistic analysis results of relationship between UA and recovery of LVEF. BMI, body mass index; LVEF, left ventricle ejection fraction; UA, uric acid; NT-proBNP, N-terminal pro brain natriuretic peptide; eGFR, estimated glomerular filtration rate; HFrEF, heart failure with reduced ejection fraction; HFrecEF, heart failure with preserved ejection fraction.

In order to assess if hyperuricemia is related with the recovery of LVEF in HFrEF patients, we divided the patients into persistent HFrEF group (45, 44.1%) and heart failure with recovery ejection fraction (HFrecEF) group (57, 49%) according to their baseline LVEF and 1 year follow-up LVEF (Supplementary Figure S1). The baseline characteristics of the two groups were shown in Table 2. The persistent HFrEF group had higher UA (358.76 vs. 307.45; *P* = 0.004) than HFrecEF group. While no significant difference was identified at baseline value of common echocardiographic parameters (Supplementary Figure S3) between two groups, HFrecEF group showed significantly increased LVEF and LVFS and significantly decreased LV end-diastolic diameter (LVEDD), LV end-systolic diameter (LVESD), LV end-diastolic volume (LVEDV), LV end- systolic volume (LVESV) after 1 year evidence-dependent medical treatment. Similar with previous studies, our study demonstrated that the HFrEF patients had higher mortality compared with HFrecEF patients (HR = 2.31, log-rank *P* = 0.037, Figure 4A).

**Table 2:** Baseline characteristics of the persistent HFrEF and HFrecEF.

**Characteristics**	**Total (*n* = 102)**	**Persistent HFrEF (*n* = 45)**	**HFrecEF (*n* = 57)**	***P*-value**
**Demographics**				
Age, years	61.13 ± 9.14	59.69 ± 8.493	62.26 ± 9.540	0.159
Male, %	63 (61.7)	34 (75.6)	29 (50.9)	0.011
BMI, kg/m^2^	23.32 ± 3.37	24.09 ± 3.396	22.74 ± 3.267	0.685
**Etiology, %**				0.160
Dilated cardiomyopathy	60 (58.8)	23 (51.1)	37 (64.9)	
Other	42 (41.2)	22 (48.9)	20 (35.1)	
**Medical history, %**				
Hypertension	33 (32.35)	15 (33.3)	19 (33.3)	0.872
Diabetes mellitus	11 (10.8)	6 (13.3)	5 (8.7)	0.461
CAD	36 (35.3)	19 (42.2)	17 (29.8)	0.193
Smoking	54 (52.9)	29 (64.4)	25 (43.9)	0.039
**Laboratory data**				
Scr, umol/L	78.56 ± 15.51	80.73 ± 13.87	76.85 ± 16.61	0.210
BUN mmol/L	6.97 ± 2.11	6.51 ± 1.88	7.28 ± 2.22	0.084
UA, μmmol/L	330.08 ± 89.62	358.76 ± 91.95	307.45 ± 81.60	0.004
eGFR, ml/min/1.73 m^2^	71.70 ± 20.98	75.70 ± 20.35	68.54 ± 21.10	0.087
NT-proBNP, ng/L	2,563.47 ± 3,036.51	2,729.10 ± 3,253.85	2,432.69 ± 2,877.4	0.629
QRS, ms	126.26 ± 25.74	126.63 ± 25.21	126.04 ± 26.29	0.920
**Echocardiographic data**				
LV EDD, mm	71.44 ± 7.92	72.71 ± 6.95	70.44 ± 8.53	0.151
LV ESD, mm	59.75 ± 7.85	60.58 ± 6.69	59.09 ± 8.66	0.344
LVEF, %	31.56 ± 7.85	32.97 ± 4.49	30.44 ± 6.21	0.023
Follow-up time, months	33.44 ± 8.67	31.04 ± 8.33	35.33 ± 8.54	0.012
**Medication use, %**				
ACE inhibitor	89 (87.3)	41 (91.1)	48 (84.2)	0.299
ARB	6 (5.8)	1 (2.2)	5 (8.8)	0.163
β-blocker	91 (89.2)	39 (86.7)	52 (91.2)	0.461
Aldosterone receptor antagonist	80 (78.4)	37 (82.2)	43 (75.4)	0.408
**NYHA, %**				0.723
I	17 (16.7)	9 (20.0)	8 (14.3)	
II	58 (56.9)	24 (53.3)	34 (60.7)	
III	23 (22.5)	10 (22.2)	13 (23.2)	
IV	3 (2.9)	2 (4.4)	1 (1.8)	
All-cause mortality	26 (25.5)	15 (33.3)	11 (19.3)	0.106
Rehospitalization	59 (60.2)	21 (50.0)	38 (67.9)	0.074
Composite outcomes events	70 (68.6)	27 (60.0)	43 (75.4)	0.095

*BMI, body mass index; CAD, coronary artery disease; Scr, serum creatinine; BUN, blood urea nitrogen; UA, Uric Acid; eGFR, estimated glomerular filtration rate; NT-pro BNP, N-terminal B-type natriuretic peptide; LVEDD, left ventricular end-diastolic diameter; LVESD, left ventricular end-systolic diameter; LVEF, left ventricular ejection fraction; ACE, angiotensin converting enzyme; ARB, angiotensin receptor blocker*.

*Values are percent or means ± SD*.

Our results displayed that initial UA value had no statistically significant correlation with LVEF at baseline (*r* = −0.11, *P* = 0.261; Figure 4B), but a significantly negative correlation with LVEF at follow-up (*r* = −0.19; *P* = 0.046, Figure 4C). And the receiver operating characteristic curves (ROC) showed that baseline UA cutoff of 312.5 μmmol/L had a sensitivity of 66.7%, specificity of 35.1%, and accuracy of 0.668 (95% CI, 0.561–0.775; *P* = 0.006) for predicting the LVEF recovery during follow-up (Figure 4D).

Multi-logistic regression analysis (Figure 4E) revealed that the likelihood of LVEF recovery at follow-up decreased by 0.6% for each unit increase of baseline UA (OR = 0.994; 95% CI, 0.988–0.999; *P* = 0.03; Figure 4E).

### Network-Based Validation of Significant Disease Relationship Between Hyperuricemia and HFrEF

**Figure 5:**
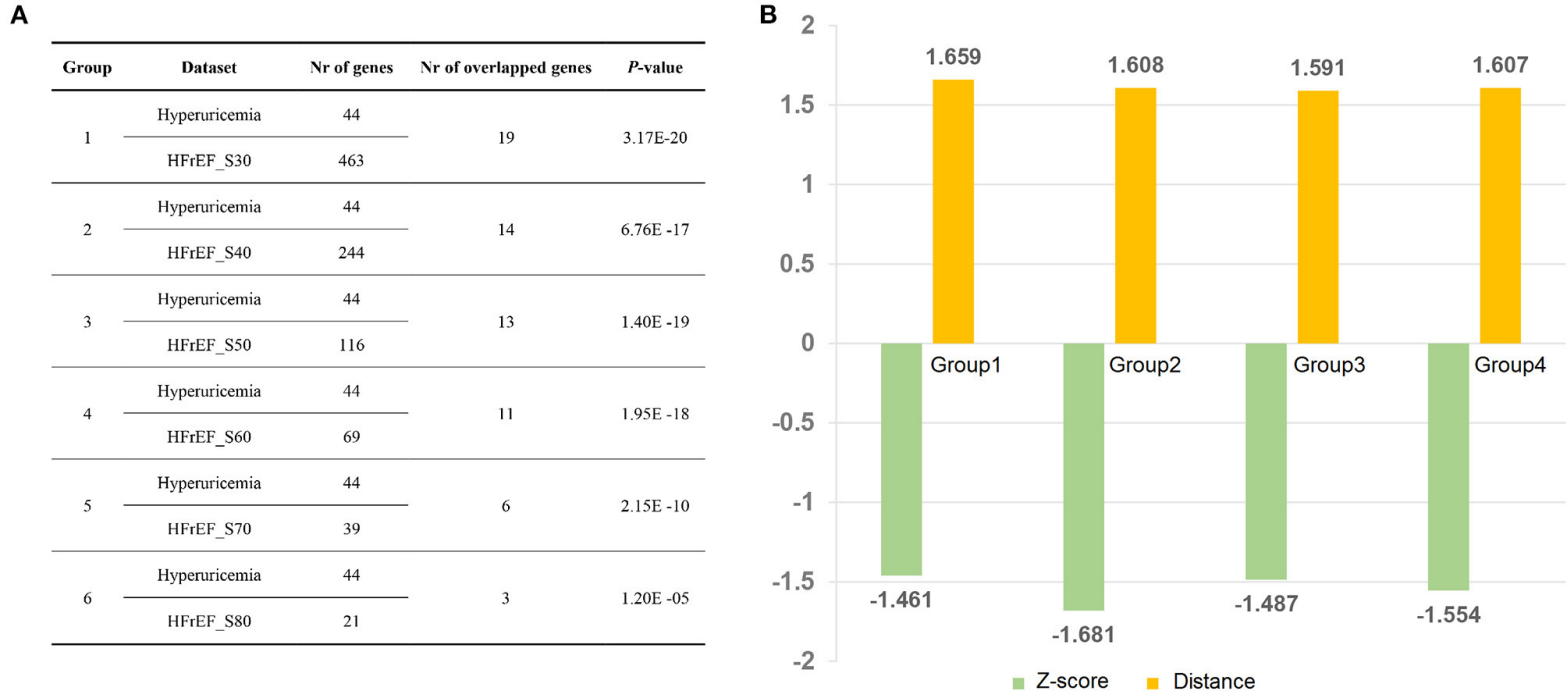
Disease relationship between Hyperuricemia and HFrEF predicted by Fisher's test and network proximity approaches. **(A)** Correlation analysis of Hyperuricemia and HFrEF gene datasets. **(B)** Z-score and distance values of four group datasets. Nr of genes indicates number of genes in each dataset; Nr of overlapped genes denotes number of overlapped genes between each two datasets; Group 1: Hyperuricemia vs. HFrEF_S30; Group 2: Hyperuricemia vs. HFrEF_S40; Group 3: Hyperuricemia vs. HFrEF_S50; Group 4: Hyperuricemia vs. HFrEF_S60.

We performed gene overlap analysis to identify the potential mechanistic relationship between hyperuricemia and HFrEF. Gene sets from each HFrEF patients exhibited high correlation with gene sets from hyperuricemia (*P* < 0.05, Figure 5A). The largest number of overlapped genes were identified between hyperuricemia and HFrEF_S30 (*n* = 19, *P* = 3.17E-20, Fisher's exact test), followed by hyperuricemia vs. HFrEF_S40 (*n* = 14, *P* = 6.76E-17, Fisher's exact test) and Hyperuricemia vs. HFrEF_S50 (*n* = 13, *P* = 1.40E-19, Fisher's exact test). The most common overlapped genes are: GAA, UMOD, APOE, LEP, MTHFR, APOA1, XDH, CRP, IL10, INS, ALDH2, KCNQ1, ALB, PPARG, REN, GPT, ESR1, NLRP3, TNF. Moreover, the *p*-value of hyperuricemia vs. HFrEF_S50 is 1.95E-18, with the overlapped gene number of 11.

We also found a strong correlation between hyperuricemia and HFrEF using network proximity approach, which proved the robustness of our result. As illustrated in Figure 5B, all the four hyperuricemia-HFrEF pairs show statistical significance (adjust *P* < 0.05). Taken together, these data verified the significant correlations between hyperuricemia and HFrEF diseases, which deserves further investigation.

### Network-Based Elucidation of Underlying Mechanistic Relationship Between Hyperuricemia and HFrEF

**Figure 6:**
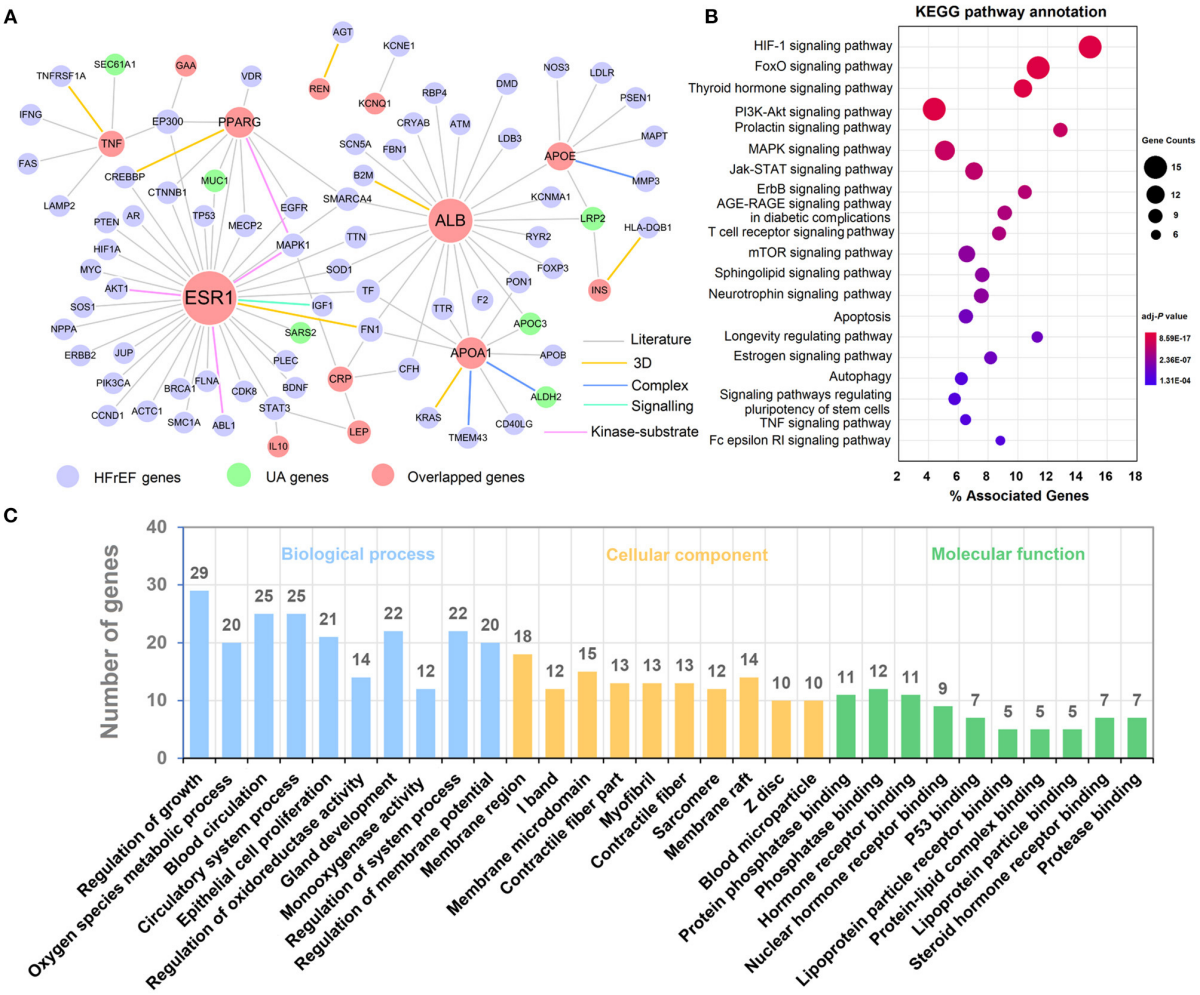
Underlying mechanisms exploration between Hyperuricemia and HFrEF. **(A)** A protein-protein interaction (PPI) network of overlapped genes. **(B)** KEGG pathways annotation results. **(C)** Biological process, cellular component and molecular function enrichment results. The label font size and node size are proportional to degree.

To explore the potential mechanistic relationship between hyperuricemia and HFrEF, we developed a protein-protein interaction (PPI) network of 14 overlapped disease genes (HFrEF_S40) based on a comprehensive human protein interactome database (14). The PPI network contains 117 edges interacting with 65 HFrEF genes and 6 hyperuricemia genes (Figure 6A). Among them, ESR1 has the most frequent gene connections (*D* = 36), followed by ALB (*D* = 24) and APOA1 (*D* = 11), indicating their key roles in both hyperuricemia and HFrEF. We further performed KEGG pathway (Figure 6B) and gene oncology enrichment (Figure 6C) analysis based on the genes within the PPI network. We identified many upregulated biological processes that were associated with blood microparticle and hormone receptor binding, including reactive oxygen species metabolic process (Figure 6C).

## Discussion

UA, a product of the metabolic breakdown of purine nucleotides, serves as a predictor of morbidity and mortality in chronic HF (6–8), as well as acute HF (9, 10). However, the predictive value of UA for LVEF recovery and prognosis in HFrEF patients have not been evaluated by evidence-based medical regimen. In present study, we demonstrated that HFrEF patients with lower baseline UA were more likely to undergo subsequent recovery of LVEF. We also showed that the higher serum UA was related to higher risk for the all-cause mortality, readmission and combined all-cause death or readmission in HF patients. These results were independent of other established risk factors such as renal function, LVEF, diuretic use, age, and NYHA functional class.

Alberto et al. previously demonstrated that in patients with acute HFrEF, univariate analysis found that hyperuricemia were associated with heart failure hospitalization or death, however, after adjustment, hyperuricemia were not associated with heart failure hospitalization or death (15). Conversely, in patients with HFpEF, hyperuricemia was the only significant predictor of the primary end point both in univariate and multivariate analyses (15). Additionally, UA was inversely correlated with the left ventricular systolic function (16). In the present study, we demonstrated in chronic stable HFrEF patients the hyperuricemia was associated with heart readmission and death. We speculate that this difference may be due to the different study populations.

However, these associations were not evaluated in HFrecEF. Reverse cardiac remodeling and LVEF recovery after the evidence-based medical therapies has become a major goal of contemporary medical and device treatment (17). While optimal pharmacologic therapies can reverse the cardiac remodeling and improve clinical outcomes, the degree of reverse remodeling is extremely variable. Some patients might not have any reverse remodeling and suffer persistent LVEF reduction and heart failure phenotype after standard treatment (4, 18). HFrEF has a spectrum range of different cellular, molecular and anatomic changes, which means HFrEF patients can exhibit different process of reverse LV remodeling. Part of them have completely myocardial recovery with increased LVEF, decreased LV volume and mass, whereas others might not have any reverse remodeling with persistently reduced EF (19). Our results are consistent with this point of view. Furthermore, we demonstrate that HFrEF patients have a wide spectrum of UA values, which may cause by tremendous interpatient heterogeneity of uric acid metabolism. The measurement of UA can help us to identify HFrEF patients with a potential possibility of LVEF recovery.

The mechanism by which UA affects the LVEF recovery and long-term prognosis of HFrEF patients remains unclear. Our gene network-based results revealed the strong correlation between HFrEF and hyperuricemia, and further mechanism exploration analysis indicated that disease-related genes were involved with multiple inflammation pathways (e.g., autophagy and TNF signaling pathway) and biological processes (e.g., reactive oxygen species metabolic process and hormone receptor). Accumulating evidence demonstrated that the management of urate-induced inflammasome or augmentation of autophagy may provide the novel effective therapies for hyperuricemia (20). Moreover, sodium-glucose cotransporter 2 inhibitors could induce transcriptional reprogramming of cardiomyocytes and activate the housekeeping pathway of autophagy, thereby acting as neurohormonal antagonists in the treatment of HFrEF (21).

Our function enrichment analyses showed that reactive oxygen species metabolic process and hormone receptor play a vital role in HFrEF. The derivative of reactive oxygen metabolites (DROM) production in the coronary circulation had an association with HFrEF development, and the DROM measurements benefited for the risk stratification of HFrEF patients (22). Emerging *in vivo* studies implicated that continuous infusion of corticotropin releasing hormone receptor 2 (Crhr2) agonist could reduce LVEF in mice, suggesting that Crhr2 blockade is a potential therapeutic strategy for patients with chronic HF (23). Additionally, blockade of mineralocorticoid receptor (hormone receptor) was able to suppress elevated uric acid and glycogen synthase kinase-3 (24).

In the human body, UA is the end production of metabolic break up of purine nucleotides. Xanthine oxidase (XO) and xanthine dehydrogenase are two enzymes responsible for the UA metabolism and production. In chronic HF patients, UA metabolism is upregulated due to higher activity of XO and growing substrates of XO (25). Meanwhile, reactive oxygen species (ROS, one item of BPs) and the hydroxyl radical are produced. These free radicals further lead to decreased nitric oxide release and increase oxidative stress synthesis (26, 27). Consequently, inflammatory pathways (e.g., TNF signaling pathway) and production of cytokines are enhanced by the imbalance of oxidative stress and decreased nitric oxide (28). Reactive oxygen and cytokines could affect clinical characters by damaging the systolic and diastolic energetics, depleting adenosine triphosphate of the sarcoplasmic reticulum, and decreasing the anaerobic threshold (29).

However, our work still has several limitations. Firstly, the sample size of Xianyang Chronic Heart Failure Prospective Cohort was small and the conclusion should be interpretated in caution in the clinical practice. Secondly, this is a prospective cohort study, some unmeasured factors such as UA-lowering agents usage, insulin resistance, peripheral vascular disease, postmenopausal state, peripheral vascular disease might influence adverse outcomes. Thirdly, the predicted mechanistic associations between hyperuricemia and HFrEF need to be validated in appropriate model systems.

## Conclusion

In HFrEF patients, a lower UA predicts the recovery of LVEF during follow-up, whereas a higher UA can be used as a predictor of long-term adverse outcome events. Gene overlap and protein-protein interaction network analyses provided a potential mechanistic relationship between hyperuricemia and HFrEF. In summary, this study offers a comprehensive framework to explore disease relationship from population and network-based perspectives.

## Data Availability Statement

The raw data supporting the conclusions of this article will be made available by the authors, without undue reservation.

## Ethics Statement

The studies involving human participants were reviewed and approved by First Affiliated Hospital of Xi'an Jiaotong University. The patients/participants provided their written informed consent to participate in this study.

## Author Contributions

XW conceived the study, analyzed the data, and wrote the manuscript. XF provided the data, analyzed the data, and revised the manuscript. QW and JF performed network analysis. LW, JL, XL, GG, DY, XB, YX, and TH revised the manuscript and reviewed the results. AM revised the manuscript and provided comments of this research. SS, JW and JF revised the manuscript and provided guidance for this study. All authors contributed to the article and approved the submitted version.

## Funding

XW was funded by the Science and Technology Talents Support Program of Shaanxi Provincial People's Hospital (2021-JY-06), Natural Science Basic Research Program of Shaanxi Province (2022JQ-855), and DY was supported by the Natural Science Foundation of Zhejiang Province, China (LQ20H020007).

## Conflict of Interest

The authors declare that the research was conducted in the absence of any commercial or financial relationships that could be construed as a potential conflict of interest.

## Publisher's Note

All claims expressed in this article are solely those of the authors and do not necessarily represent those of their affiliated organizations, or those of the publisher, the editors and the reviewers. Any product that may be evaluated in this article, or claim that may be made by its manufacturer, is not guaranteed or endorsed by the publisher.
