# Supplementary material for: Uric Acid Predicts Recovery of Left Ventricular Function and Adverse Events in Heart Failure With Reduced Ejection Fraction: Potential Mechanistic Insight From Network Analyses
Source: Front Cardiovasc Med. 2022 Jul 15;9:853870. doi: 10.3389/fcvm.2022.853870 (PMC9334530; doi:10.3389/fcvm.2022.853870)
Supplement: Supplementary file 1 [file Data_Sheet_1.docx]

Table S1. Criteria to determine presence of diseases in NIS database

| Diseases | Diagnoses code |
| --- | --- |
| Coagulopathy | ICD-10-CM diagnoses code D684, D68311, D6851, D68312, D6861, D689, D6832, D682, D681, D6862, D68318, D6859, D688, D6869, D6852, D680 |
| Obesity | ICD-10-CM diagnoses code E669, E668, E6609, E661, E6601, E662 |
| Hypertension | ICD-10-CM diagnoses code I10, I152, I151, I158, I150, I159 |
| Hypothyroidism | ICD-10-CM diagnoses code E034, E030, E031, E032, E039, E034, E038, E033 |
| Coronary artery disease | ICD-10-CM diagnoses code I25111, I25118, I25119, I25110, I2510, I252, I255, I256, I25812, I25810, I25811, I2582, I2584, I2583, I2589, I259 |
| Atrial fibrillation | ICD-10-CM diagnoses code I482, I4820, I4811, I4819, I480, I4821, I481, I4891 |
| Diabetes mellitus | ICD-10-CM diagnoses code E10.x-E13.x, |
| Peripheral vascular disease | ICD-10-CM diagnoses code I7389, I739, I70201, I70209, I70503, I70502, I70508, I70501, I70509, I70603, I70602, I70608, I70601, I70609, I70703, I70702, I70708, I70701, I70709, I70303, I70302, I70308, I70301, I70309 |
| Hypercholesteremia | ICD-10-CM diagnoses code E7801, E780, E7800 |
| Alcohol use | ICD-10-CM diagnoses code F10180, F1014, F10150, F10151, F10159, F10181, F10182, F10121, F10120, F10129, F10188, F1019, F10131, F10132, F10130, F10139, F1011, F1010, F10280, F1024, F1026, F1027, F10250, F10251, F10259, F10281, F10282, F10221, F10220, F10229, F10288, F1029, F10231, F10232, F10230, F10239, F1021, F1020, F10980, F1094, F1096, F1097, F10950, F10951, F10959, F10981, F10982, F10921, F10920, F10929, F10988, F1099, F10931, F10932, F10930, F10939 |
| Tobacco abuse | ICD-10-CM diagnoses code Z716, Z720, F17221, F17220, F17228, F17229, F17223, F17291, F17290, F17298, F17299, F17293 |
| Acute respiratory failure | ICD-10-CM diagnoses code J9622, J9621, J9620, J9602, J9601, J9600, J9692, J9691, J9690 |
| Acute renal failure | ICD-10-CM diagnoses code N171, N172, N170, N179, N178, N19, N990, N178 |
| Acute hepatic failure | ICD-10-CM diagnoses code K7201, K7200, K7291, K7290 |
| Acute pulmonary edema | ICD-10-CM diagnoses code J810 |
| Hematodialysis | ICD-10-PCS codes 5A1D00Z, 5A1D60Z, 5A1D70Z, 5A1D80Z, 5A1D90Z |
| Ventricular fibrillation | ICD-10-CM diagnoses code I4901 |
| Cardiac shock | ICD-10-CM diagnoses code R570 |
| Sudden cardiac arrest | ICD-10-CM diagnoses code Z8674 |
| Cardio-pulmonary resuscitation | ICD-10-PCS codes 5A12012 |
| Ventilator use | ICD-10-PCS codes 5A1935Z, 5A1945Z, 5A1955Z |
| LVAD | ICD-10-PCS codes 02HA0QZ |
| IABP | ICD-10-PCS codes 5A02210 |
| ECMO | ICD-10-PCS codes 5A15223, 5A1522F, 5A1522G, 5A1522H |
| PCI | ICD-10-PCS codes 02703ZZ, 02704ZZ, 02713ZZ, 02714ZZ, 02723ZZ, 02724ZZ, 02733ZZ, 02734ZZ, 02Q03ZZ, 02Q04ZZ, 02Q13ZZ, 02Q14ZZ, 02Q23ZZ, 02Q24ZZ, 02Q33ZZ, 02Q34ZZ, 0270346, 027034Z, 0270356, 027035Z, 0270366, 027036Z, |
| Permanent pacemaker implantation | ICD-10-PCS codes 02HK3JZ, 02HN0JZ, 02H60JZ, 02H60NZ, 02H63NZ, 02H64JZ, 02H64NZ, 02HK0JZ, 02HK0NZ, 02HK3NZ, 02HK4JZ, 02HK4NZ, 02HN4JZ, 0JH604Z, 0JH634Z, 0JH605Z, 0JH607Z, 0JH635Z, 0JH606Z, 0JH636Z, 0JH637Z |
| Hyperuricemia | ICD-10-CM diagnoses code E790 |
| Fluid and electrolyte disorders | ICD-10-CM diagnoses code E872, E873, E8770, E875, E870, E876, E871, E874, E878, E8779, E8771 |
| Valvular heart disease | ICD-10-CM diagnoses code I058, I051, I050, I052, I059, I068, I061, I060, I062, I069, I078, I071, I070, I072, I079, I083, I088, I082, I080, I081, I089, I351, I350, I352, I359, I358, I351, I350, I352, I359, I358, I361, I360, I362, I369, I368, I379, I371, I370, I372, I378 |
| Chronic kidney disease | ICD-10-CM diagnoses code N181, N182, N183, N1830, N1831, N1832, N184, N185, N189, N186 |
| Chronic liver disease | ICD-10-CM diagnoses code K7211, K7210' |
| Chronic lung disease | ICD-10-CM diagnoses code B63, I2782, I2781, I2783, I2789, I279, J40, J418, J411, J410, J42, J432, J439, J438, J431, J430, J441, J440, J449, J668, J660, J662, J661, J677, J671, J672, J670, J678, J679, J674, J676, J675, J673 |
| Chronic anemia | ICD-10-CM diagnoses code D500, D509, D508, D501, D513, D518, D512, D510, D511, D519, D520, D521, D529, D528, D539, D531, D538, D530, D532 |
| Carotid artery disease | ICD-10-CM diagnoses code I6523, I6522, I6521, I6529 |
| Peripheral vascular disease | ICD-10-CM diagnoses code I70201, I70209, I70503, I70502, I70508, I70501, I70509, I70603, I70602, I70608, I70601, I70609, I70703, I70702, I70708, I70701, I70709, I70303, I70302, I70308, I70301, I70309, I7389, I739 |
| Long term (current) use of oral hypoglycemic drugs | ICD-10-CM diagnoses code Z7984 |
| Prior MI | ICD-10-CM diagnoses code I252 |
| Prior PCI | ICD-10-CM diagnoses code Z955, Z9861 |
| Prior CABG | ICD-10-CM diagnoses code Z951, Z95818, Z959, Z955 |
| Prior stroke | ICD-10-CM diagnoses code Z8673 |
| Prior cardiac pacemaker | ICD-10-CM diagnoses code Z950 |
| Prior LVAD | ICD-10-CM diagnoses code Z95811 |

CABG indicates coronary artery bypass grafting; LVAD, left ventricular assist device; PCI, percutaneous coronary intervention; MI, myocardial infarction; ECHO, extracorporeal membrane oxygenation.

Table S2. HFrEF genes via searching the terms “Heart failure with a reduced ejection fraction” in GeneCards (https://www.genecards.org/) database and reserved those HFrEF genes with score value higher than 30.

| Gene set | Gene Entrez ID | Gene Symbol |
| --- | --- | --- |
| HFrEF_S80 | 7273 | TTN |
| HFrEF_S80 | 6331 | SCN5A |
| HFrEF_S80 | 4000 | LMNA |
| HFrEF_S80 | 1482 | NKX2-5 |
| HFrEF_S80 | 1832 | DSP |
| HFrEF_S80 | 1636 | ACE |
| HFrEF_S80 | 4625 | MYH7 |
| HFrEF_S80 | 7157 | TP53 |
| HFrEF_S80 | 7124 | TNF |
| HFrEF_S80 | 5339 | PLEC |
| HFrEF_S80 | 3569 | IL6 |
| HFrEF_S80 | 2626 | GATA4 |
| HFrEF_S80 | 6261 | RYR1 |
| HFrEF_S80 | 4624 | MYH6 |
| HFrEF_S80 | 5781 | PTPN11 |
| HFrEF_S80 | 4879 | NPPB |
| HFrEF_S80 | 3630 | INS |
| HFrEF_S80 | 2697 | GJA1 |
| HFrEF_S80 | 4607 | MYBPC3 |
| HFrEF_S80 | 3784 | KCNQ1 |
| HFrEF_S80 | 5728 | PTEN |
| HFrEF_S70 | 7273 | TTN |
| HFrEF_S70 | 6331 | SCN5A |
| HFrEF_S70 | 4000 | LMNA |
| HFrEF_S70 | 1482 | NKX2-5 |
| HFrEF_S70 | 1832 | DSP |
| HFrEF_S70 | 1636 | ACE |
| HFrEF_S70 | 4625 | MYH7 |
| HFrEF_S70 | 7157 | TP53 |
| HFrEF_S70 | 7124 | TNF |
| HFrEF_S70 | 5339 | PLEC |
| HFrEF_S70 | 3569 | IL6 |
| HFrEF_S70 | 2626 | GATA4 |
| HFrEF_S70 | 6261 | RYR1 |
| HFrEF_S70 | 4624 | MYH6 |
| HFrEF_S70 | 5781 | PTPN11 |
| HFrEF_S70 | 4879 | NPPB |
| HFrEF_S70 | 3630 | INS |
| HFrEF_S70 | 2697 | GJA1 |
| HFrEF_S70 | 4607 | MYBPC3 |
| HFrEF_S70 | 3784 | KCNQ1 |
| HFrEF_S70 | 5728 | PTEN |
| HFrEF_S70 | 7139 | TNNT2 |
| HFrEF_S70 | 4878 | NPPA |
| HFrEF_S70 | 3586 | IL10 |
| HFrEF_S70 | 51422 | PRKAG2 |
| HFrEF_S70 | 1756 | DMD |
| HFrEF_S70 | 348 | APOE |
| HFrEF_S70 | 7137 | TNNI3 |
| HFrEF_S70 | 5428 | POLG |
| HFrEF_S70 | 6910 | TBX5 |
| HFrEF_S70 | 213 | ALB |
| HFrEF_S70 | 2627 | GATA6 |
| HFrEF_S70 | 5594 | MAPK1 |
| HFrEF_S70 | 3845 | KRAS |
| HFrEF_S70 | 675 | BRCA2 |
| HFrEF_S70 | 673 | BRAF |
| HFrEF_S70 | 6262 | RYR2 |
| HFrEF_S70 | 7040 | TGFB1 |
| HFrEF_S70 | 4846 | NOS3 |
| HFrEF_S60 | 7273 | TTN |
| HFrEF_S60 | 6331 | SCN5A |
| HFrEF_S60 | 4000 | LMNA |
| HFrEF_S60 | 1482 | NKX2-5 |
| HFrEF_S60 | 1832 | DSP |
| HFrEF_S60 | 1636 | ACE |
| HFrEF_S60 | 4625 | MYH7 |
| HFrEF_S60 | 7157 | TP53 |
| HFrEF_S60 | 7124 | TNF |
| HFrEF_S60 | 5339 | PLEC |
| HFrEF_S60 | 3569 | IL6 |
| HFrEF_S60 | 2626 | GATA4 |
| HFrEF_S60 | 6261 | RYR1 |
| HFrEF_S60 | 4624 | MYH6 |
| HFrEF_S60 | 5781 | PTPN11 |
| HFrEF_S60 | 4879 | NPPB |
| HFrEF_S60 | 3630 | INS |
| HFrEF_S60 | 2697 | GJA1 |
| HFrEF_S60 | 4607 | MYBPC3 |
| HFrEF_S60 | 3784 | KCNQ1 |
| HFrEF_S60 | 5728 | PTEN |
| HFrEF_S60 | 7139 | TNNT2 |
| HFrEF_S60 | 4878 | NPPA |
| HFrEF_S60 | 3586 | IL10 |
| HFrEF_S60 | 51422 | PRKAG2 |
| HFrEF_S60 | 1756 | DMD |
| HFrEF_S60 | 348 | APOE |
| HFrEF_S60 | 7137 | TNNI3 |
| HFrEF_S60 | 5428 | POLG |
| HFrEF_S60 | 6910 | TBX5 |
| HFrEF_S60 | 213 | ALB |
| HFrEF_S60 | 2627 | GATA6 |
| HFrEF_S60 | 5594 | MAPK1 |
| HFrEF_S60 | 3845 | KRAS |
| HFrEF_S60 | 675 | BRCA2 |
| HFrEF_S60 | 673 | BRAF |
| HFrEF_S60 | 6262 | RYR2 |
| HFrEF_S60 | 7040 | TGFB1 |
| HFrEF_S60 | 4846 | NOS3 |
| HFrEF_S60 | 1401 | CRP |
| HFrEF_S60 | 2260 | FGFR1 |
| HFrEF_S60 | 2200 | FBN1 |
| HFrEF_S60 | 1674 | DES |
| HFrEF_S60 | 4524 | MTHFR |
| HFrEF_S60 | 5290 | PIK3CA |
| HFrEF_S60 | 7276 | TTR |
| HFrEF_S60 | 3553 | IL1B |
| HFrEF_S60 | 2316 | FLNA |
| HFrEF_S60 | 3123 | HLA-DRB1 |
| HFrEF_S60 | 5468 | PPARG |
| HFrEF_S60 | 5972 | REN |
| HFrEF_S60 | 2261 | FGFR3 |
| HFrEF_S60 | 3717 | JAK2 |
| HFrEF_S60 | 3458 | IFNG |
| HFrEF_S60 | 3479 | IGF1 |
| HFrEF_S60 | 4204 | MECP2 |
| HFrEF_S60 | 5894 | RAF1 |
| HFrEF_S60 | 9464 | HAND2 |
| HFrEF_S60 | 25 | ABL1 |
| HFrEF_S60 | 1906 | EDN1 |
| HFrEF_S60 | 2099 | ESR1 |
| HFrEF_S60 | 1499 | CTNNB1 |
| HFrEF_S60 | 2147 | F2 |
| HFrEF_S60 | 185 | AGTR1 |
| HFrEF_S60 | 153 | ADRB1 |
| HFrEF_S60 | 5663 | PSEN1 |
| HFrEF_S60 | 70 | ACTC1 |
| HFrEF_S60 | 183 | AGT |
| HFrEF_S60 | 4838 | NODAL |
| HFrEF_S50 | 7273 | TTN |
| HFrEF_S50 | 6331 | SCN5A |
| HFrEF_S50 | 4000 | LMNA |
| HFrEF_S50 | 1482 | NKX2-5 |
| HFrEF_S50 | 1832 | DSP |
| HFrEF_S50 | 1636 | ACE |
| HFrEF_S50 | 4625 | MYH7 |
| HFrEF_S50 | 7157 | TP53 |
| HFrEF_S50 | 7124 | TNF |
| HFrEF_S50 | 5339 | PLEC |
| HFrEF_S50 | 3569 | IL6 |
| HFrEF_S50 | 2626 | GATA4 |
| HFrEF_S50 | 6261 | RYR1 |
| HFrEF_S50 | 4624 | MYH6 |
| HFrEF_S50 | 5781 | PTPN11 |
| HFrEF_S50 | 4879 | NPPB |
| HFrEF_S50 | 3630 | INS |
| HFrEF_S50 | 2697 | GJA1 |
| HFrEF_S50 | 4607 | MYBPC3 |
| HFrEF_S50 | 3784 | KCNQ1 |
| HFrEF_S50 | 5728 | PTEN |
| HFrEF_S50 | 7139 | TNNT2 |
| HFrEF_S50 | 4878 | NPPA |
| HFrEF_S50 | 3586 | IL10 |
| HFrEF_S50 | 51422 | PRKAG2 |
| HFrEF_S50 | 1756 | DMD |
| HFrEF_S50 | 348 | APOE |
| HFrEF_S50 | 7137 | TNNI3 |
| HFrEF_S50 | 5428 | POLG |
| HFrEF_S50 | 6910 | TBX5 |
| HFrEF_S50 | 213 | ALB |
| HFrEF_S50 | 2627 | GATA6 |
| HFrEF_S50 | 5594 | MAPK1 |
| HFrEF_S50 | 3845 | KRAS |
| HFrEF_S50 | 675 | BRCA2 |
| HFrEF_S50 | 673 | BRAF |
| HFrEF_S50 | 6262 | RYR2 |
| HFrEF_S50 | 7040 | TGFB1 |
| HFrEF_S50 | 4846 | NOS3 |
| HFrEF_S50 | 1401 | CRP |
| HFrEF_S50 | 2260 | FGFR1 |
| HFrEF_S50 | 2200 | FBN1 |
| HFrEF_S50 | 1674 | DES |
| HFrEF_S50 | 4524 | MTHFR |
| HFrEF_S50 | 5290 | PIK3CA |
| HFrEF_S50 | 7276 | TTR |
| HFrEF_S50 | 3553 | IL1B |
| HFrEF_S50 | 2316 | FLNA |
| HFrEF_S50 | 3123 | HLA-DRB1 |
| HFrEF_S50 | 5468 | PPARG |
| HFrEF_S50 | 5972 | REN |
| HFrEF_S50 | 2261 | FGFR3 |
| HFrEF_S50 | 3717 | JAK2 |
| HFrEF_S50 | 3458 | IFNG |
| HFrEF_S50 | 3479 | IGF1 |
| HFrEF_S50 | 4204 | MECP2 |
| HFrEF_S50 | 5894 | RAF1 |
| HFrEF_S50 | 9464 | HAND2 |
| HFrEF_S50 | 25 | ABL1 |
| HFrEF_S50 | 1906 | EDN1 |
| HFrEF_S50 | 2099 | ESR1 |
| HFrEF_S50 | 1499 | CTNNB1 |
| HFrEF_S50 | 2147 | F2 |
| HFrEF_S50 | 185 | AGTR1 |
| HFrEF_S50 | 153 | ADRB1 |
| HFrEF_S50 | 5663 | PSEN1 |
| HFrEF_S50 | 70 | ACTC1 |
| HFrEF_S50 | 183 | AGT |
| HFrEF_S50 | 4838 | NODAL |
| HFrEF_S50 | 7422 | VEGFA |
| HFrEF_S50 | 57057 | TBX20 |
| HFrEF_S50 | 3757 | KCNH2 |
| HFrEF_S50 | 4763 | NF1 |
| HFrEF_S50 | 4851 | NOTCH1 |
| HFrEF_S50 | 338 | APOB |
| HFrEF_S50 | 5979 | RET |
| HFrEF_S50 | 7450 | VWF |
| HFrEF_S50 | 154 | ADRB2 |
| HFrEF_S50 | 659 | BMPR2 |
| HFrEF_S50 | 5350 | PLN |
| HFrEF_S50 | 7168 | TPM1 |
| HFrEF_S50 | 56652 | TWNK |
| HFrEF_S50 | 3952 | LEP |
| HFrEF_S50 | 6647 | SOD1 |
| HFrEF_S50 | 672 | BRCA1 |
| HFrEF_S50 | 3077 | HFE |
| HFrEF_S50 | 2318 | FLNC |
| HFrEF_S50 | 207 | AKT1 |
| HFrEF_S50 | 2022 | ENG |
| HFrEF_S50 | 3265 | HRAS |
| HFrEF_S50 | 9370 | ADIPOQ |
| HFrEF_S50 | 1080 | CFTR |
| HFrEF_S50 | 7099 | TLR4 |
| HFrEF_S50 | 3557 | IL1RN |
| HFrEF_S50 | 11155 | LDB3 |
| HFrEF_S50 | 335 | APOA1 |
| HFrEF_S50 | 627 | BDNF |
| HFrEF_S50 | 3576 | CXCL8 |
| HFrEF_S50 | 291 | SLC25A4 |
| HFrEF_S50 | 3767 | KCNJ11 |
| HFrEF_S50 | 6833 | ABCC8 |
| HFrEF_S50 | 472 | ATM |
| HFrEF_S50 | 4313 | MMP2 |
| HFrEF_S50 | 88 | ACTN2 |
| HFrEF_S50 | 2335 | FN1 |
| HFrEF_S50 | 5054 | SERPINE1 |
| HFrEF_S50 | 79147 | FKRP |
| HFrEF_S50 | 775 | CACNA1C |
| HFrEF_S50 | 2006 | ELN |
| HFrEF_S50 | 5318 | PKP2 |
| HFrEF_S50 | 1956 | EGFR |
| HFrEF_S50 | 1493 | CTLA4 |
| HFrEF_S50 | 79188 | TMEM43 |
| HFrEF_S50 | 2033 | EP300 |
| HFrEF_S50 | 4318 | MMP9 |
| HFrEF_S50 | 282996 | RBM20 |
| HFrEF_S40 | 7273 | TTN |
| HFrEF_S40 | 6331 | SCN5A |
| HFrEF_S40 | 4000 | LMNA |
| HFrEF_S40 | 1482 | NKX2-5 |
| HFrEF_S40 | 1832 | DSP |
| HFrEF_S40 | 1636 | ACE |
| HFrEF_S40 | 4625 | MYH7 |
| HFrEF_S40 | 7157 | TP53 |
| HFrEF_S40 | 7124 | TNF |
| HFrEF_S40 | 5339 | PLEC |
| HFrEF_S40 | 3569 | IL6 |
| HFrEF_S40 | 2626 | GATA4 |
| HFrEF_S40 | 6261 | RYR1 |
| HFrEF_S40 | 4624 | MYH6 |
| HFrEF_S40 | 5781 | PTPN11 |
| HFrEF_S40 | 4879 | NPPB |
| HFrEF_S40 | 3630 | INS |
| HFrEF_S40 | 2697 | GJA1 |
| HFrEF_S40 | 4607 | MYBPC3 |
| HFrEF_S40 | 3784 | KCNQ1 |
| HFrEF_S40 | 5728 | PTEN |
| HFrEF_S40 | 7139 | TNNT2 |
| HFrEF_S40 | 4878 | NPPA |
| HFrEF_S40 | 3586 | IL10 |
| HFrEF_S40 | 51422 | PRKAG2 |
| HFrEF_S40 | 1756 | DMD |
| HFrEF_S40 | 348 | APOE |
| HFrEF_S40 | 7137 | TNNI3 |
| HFrEF_S40 | 5428 | POLG |
| HFrEF_S40 | 6910 | TBX5 |
| HFrEF_S40 | 213 | ALB |
| HFrEF_S40 | 2627 | GATA6 |
| HFrEF_S40 | 5594 | MAPK1 |
| HFrEF_S40 | 3845 | KRAS |
| HFrEF_S40 | 675 | BRCA2 |
| HFrEF_S40 | 673 | BRAF |
| HFrEF_S40 | 6262 | RYR2 |
| HFrEF_S40 | 7040 | TGFB1 |
| HFrEF_S40 | 4846 | NOS3 |
| HFrEF_S40 | 1401 | CRP |
| HFrEF_S40 | 2260 | FGFR1 |
| HFrEF_S40 | 2200 | FBN1 |
| HFrEF_S40 | 1674 | DES |
| HFrEF_S40 | 4524 | MTHFR |
| HFrEF_S40 | 5290 | PIK3CA |
| HFrEF_S40 | 7276 | TTR |
| HFrEF_S40 | 3553 | IL1B |
| HFrEF_S40 | 2316 | FLNA |
| HFrEF_S40 | 3123 | HLA-DRB1 |
| HFrEF_S40 | 5468 | PPARG |
| HFrEF_S40 | 5972 | REN |
| HFrEF_S40 | 2261 | FGFR3 |
| HFrEF_S40 | 3717 | JAK2 |
| HFrEF_S40 | 3458 | IFNG |
| HFrEF_S40 | 3479 | IGF1 |
| HFrEF_S40 | 4204 | MECP2 |
| HFrEF_S40 | 5894 | RAF1 |
| HFrEF_S40 | 9464 | HAND2 |
| HFrEF_S40 | 25 | ABL1 |
| HFrEF_S40 | 1906 | EDN1 |
| HFrEF_S40 | 2099 | ESR1 |
| HFrEF_S40 | 1499 | CTNNB1 |
| HFrEF_S40 | 2147 | F2 |
| HFrEF_S40 | 185 | AGTR1 |
| HFrEF_S40 | 153 | ADRB1 |
| HFrEF_S40 | 5663 | PSEN1 |
| HFrEF_S40 | 70 | ACTC1 |
| HFrEF_S40 | 183 | AGT |
| HFrEF_S40 | 4838 | NODAL |
| HFrEF_S40 | 7422 | VEGFA |
| HFrEF_S40 | 57057 | TBX20 |
| HFrEF_S40 | 3757 | KCNH2 |
| HFrEF_S40 | 4763 | NF1 |
| HFrEF_S40 | 4851 | NOTCH1 |
| HFrEF_S40 | 338 | APOB |
| HFrEF_S40 | 5979 | RET |
| HFrEF_S40 | 7450 | VWF |
| HFrEF_S40 | 154 | ADRB2 |
| HFrEF_S40 | 659 | BMPR2 |
| HFrEF_S40 | 5350 | PLN |
| HFrEF_S40 | 7168 | TPM1 |
| HFrEF_S40 | 56652 | TWNK |
| HFrEF_S40 | 3952 | LEP |
| HFrEF_S40 | 6647 | SOD1 |
| HFrEF_S40 | 672 | BRCA1 |
| HFrEF_S40 | 3077 | HFE |
| HFrEF_S40 | 2318 | FLNC |
| HFrEF_S40 | 207 | AKT1 |
| HFrEF_S40 | 2022 | ENG |
| HFrEF_S40 | 3265 | HRAS |
| HFrEF_S40 | 9370 | ADIPOQ |
| HFrEF_S40 | 1080 | CFTR |
| HFrEF_S40 | 7099 | TLR4 |
| HFrEF_S40 | 3557 | IL1RN |
| HFrEF_S40 | 11155 | LDB3 |
| HFrEF_S40 | 335 | APOA1 |
| HFrEF_S40 | 627 | BDNF |
| HFrEF_S40 | 3576 | CXCL8 |
| HFrEF_S40 | 291 | SLC25A4 |
| HFrEF_S40 | 3767 | KCNJ11 |
| HFrEF_S40 | 6833 | ABCC8 |
| HFrEF_S40 | 472 | ATM |
| HFrEF_S40 | 4313 | MMP2 |
| HFrEF_S40 | 88 | ACTN2 |
| HFrEF_S40 | 2335 | FN1 |
| HFrEF_S40 | 5054 | SERPINE1 |
| HFrEF_S40 | 79147 | FKRP |
| HFrEF_S40 | 775 | CACNA1C |
| HFrEF_S40 | 2006 | ELN |
| HFrEF_S40 | 5318 | PKP2 |
| HFrEF_S40 | 1956 | EGFR |
| HFrEF_S40 | 1493 | CTLA4 |
| HFrEF_S40 | 79188 | TMEM43 |
| HFrEF_S40 | 2033 | EP300 |
| HFrEF_S40 | 4318 | MMP9 |
| HFrEF_S40 | 282996 | RBM20 |
| HFrEF_S40 | 58 | ACTA1 |
| HFrEF_S40 | 959 | CD40LG |
| HFrEF_S40 | 2717 | GLA |
| HFrEF_S40 | 2778 | GNAS |
| HFrEF_S40 | 1282 | COL4A1 |
| HFrEF_S40 | 3481 | IGF2 |
| HFrEF_S40 | 6347 | CCL2 |
| HFrEF_S40 | 1277 | COL1A1 |
| HFrEF_S40 | 50484 | RRM2B |
| HFrEF_S40 | 6389 | SDHA |
| HFrEF_S40 | 1909 | EDNRA |
| HFrEF_S40 | 3920 | LAMP2 |
| HFrEF_S40 | 948 | CD36 |
| HFrEF_S40 | 3815 | KIT |
| HFrEF_S40 | 3119 | HLA-DQB1 |
| HFrEF_S40 | 7056 | THBD |
| HFrEF_S40 | 4306 | NR3C2 |
| HFrEF_S40 | 6774 | STAT3 |
| HFrEF_S40 | 4023 | LPL |
| HFrEF_S40 | 7421 | VDR |
| HFrEF_S40 | 5311 | PKD2 |
| HFrEF_S40 | 4508 | MT-ATP6 |
| HFrEF_S40 | 50943 | FOXP3 |
| HFrEF_S40 | 695 | BTK |
| HFrEF_S40 | 859 | CAV3 |
| HFrEF_S40 | 650 | BMP2 |
| HFrEF_S40 | 7414 | VCL |
| HFrEF_S40 | 9531 | BAG3 |
| HFrEF_S40 | 6901 | TAFAZZIN |
| HFrEF_S40 | 5444 | PON1 |
| HFrEF_S40 | 2702 | GJA5 |
| HFrEF_S40 | 3690 | ITGB3 |
| HFrEF_S40 | 5167 | ENPP1 |
| HFrEF_S40 | 5373 | PMM2 |
| HFrEF_S40 | 4633 | MYL2 |
| HFrEF_S40 | 950 | SCARB2 |
| HFrEF_S40 | 4512 | MT-CO1 |
| HFrEF_S40 | 29954 | POMT2 |
| HFrEF_S40 | 4353 | MPO |
| HFrEF_S40 | 3949 | LDLR |
| HFrEF_S40 | 7132 | TNFRSF1A |
| HFrEF_S40 | 3383 | ICAM1 |
| HFrEF_S40 | 8243 | SMC1A |
| HFrEF_S40 | 2548 | GAA |
| HFrEF_S40 | 5308 | PITX2 |
| HFrEF_S40 | 3728 | JUP |
| HFrEF_S40 | 8878 | SQSTM1 |
| HFrEF_S40 | 9927 | MFN2 |
| HFrEF_S40 | 3762 | KCNJ5 |
| HFrEF_S40 | 3558 | IL2 |
| HFrEF_S40 | 4137 | MAPT |
| HFrEF_S40 | 4519 | MT-CYB |
| HFrEF_S40 | 6469 | SHH |
| HFrEF_S40 | 2153 | F5 |
| HFrEF_S40 | 5265 | SERPINA1 |
| HFrEF_S40 | 567 | B2M |
| HFrEF_S40 | 6654 | SOS1 |
| HFrEF_S40 | 4609 | MYC |
| HFrEF_S40 | 4535 | MT-ND1 |
| HFrEF_S40 | 2263 | FGFR2 |
| HFrEF_S40 | 1024 | CDK8 |
| HFrEF_S40 | 55687 | TRMU |
| HFrEF_S40 | 595 | CCND1 |
| HFrEF_S40 | 1387 | CREBBP |
| HFrEF_S40 | 3565 | IL4 |
| HFrEF_S40 | 4843 | NOS2 |
| HFrEF_S40 | 7042 | TGFB2 |
| HFrEF_S40 | 5443 | POMC |
| HFrEF_S40 | 355 | FAS |
| HFrEF_S40 | 2253 | FGF8 |
| HFrEF_S40 | 6622 | SNCA |
| HFrEF_S40 | 2056 | EPO |
| HFrEF_S40 | 84665 | MYPN |
| HFrEF_S40 | 2776 | GNAQ |
| HFrEF_S40 | 7010 | TEK |
| HFrEF_S40 | 5727 | PTCH1 |
| HFrEF_S40 | 1829 | DSG2 |
| HFrEF_S40 | 3827 | KNG1 |
| HFrEF_S40 | 8291 | DYSF |
| HFrEF_S40 | 5950 | RBP4 |
| HFrEF_S40 | 10060 | ABCC9 |
| HFrEF_S40 | 4669 | NAGLU |
| HFrEF_S40 | 4514 | MT-CO3 |
| HFrEF_S40 | 1769 | DNAH8 |
| HFrEF_S40 | 4312 | MMP1 |
| HFrEF_S40 | 1410 | CRYAB |
| HFrEF_S40 | 462 | SERPINC1 |
| HFrEF_S40 | 3778 | KCNMA1 |
| HFrEF_S40 | 4598 | MVK |
| HFrEF_S40 | 8048 | CSRP3 |
| HFrEF_S40 | 1440 | CSF3 |
| HFrEF_S40 | 6392 | SDHD |
| HFrEF_S40 | 9421 | HAND1 |
| HFrEF_S40 | 652 | BMP4 |
| HFrEF_S40 | 2629 | GBA |
| HFrEF_S40 | 2218 | FKTN |
| HFrEF_S40 | 1029 | CDKN2A |
| HFrEF_S40 | 1376 | CPT2 |
| HFrEF_S40 | 10585 | POMT1 |
| HFrEF_S40 | 825 | CAPN3 |
| HFrEF_S40 | 7043 | TGFB3 |
| HFrEF_S40 | 3162 | HMOX1 |
| HFrEF_S40 | 3091 | HIF1A |
| HFrEF_S40 | 2064 | ERBB2 |
| HFrEF_S40 | 4893 | NRAS |
| HFrEF_S40 | 836 | CASP3 |
| HFrEF_S40 | 3753 | KCNE1 |
| HFrEF_S40 | 3559 | IL2RA |
| HFrEF_S40 | 3030 | HADHA |
| HFrEF_S40 | 920 | CD4 |
| HFrEF_S40 | 8557 | TCAP |
| HFrEF_S40 | 3606 | IL18 |
| HFrEF_S40 | 6597 | SMARCA4 |
| HFrEF_S40 | 9742 | IFT140 |
| HFrEF_S40 | 51594 | NBAS |
| HFrEF_S40 | 367 | AR |
| HFrEF_S40 | 6532 | SLC6A4 |
| HFrEF_S40 | 4540 | MT-ND5 |
| HFrEF_S40 | 57190 | SELENON |
| HFrEF_S40 | 2475 | MTOR |
| HFrEF_S40 | 4314 | MMP3 |
| HFrEF_S40 | 1278 | COL1A2 |
| HFrEF_S40 | 7076 | TIMP1 |
| HFrEF_S40 | 3075 | CFH |
| HFrEF_S40 | 7018 | TF |
| HFrEF_S40 | 1565 | CYP2D6 |
| HFrEF_S40 | 11093 | ADAMTS13 |
| HFrEF_S40 | 10269 | ZMPSTE24 |
| HFrEF_S30 | 7273 | TTN |
| HFrEF_S30 | 6331 | SCN5A |
| HFrEF_S30 | 4000 | LMNA |
| HFrEF_S30 | 1482 | NKX2-5 |
| HFrEF_S30 | 1832 | DSP |
| HFrEF_S30 | 1636 | ACE |
| HFrEF_S30 | 4625 | MYH7 |
| HFrEF_S30 | 7157 | TP53 |
| HFrEF_S30 | 7124 | TNF |
| HFrEF_S30 | 5339 | PLEC |
| HFrEF_S30 | 3569 | IL6 |
| HFrEF_S30 | 2626 | GATA4 |
| HFrEF_S30 | 6261 | RYR1 |
| HFrEF_S30 | 4624 | MYH6 |
| HFrEF_S30 | 5781 | PTPN11 |
| HFrEF_S30 | 4879 | NPPB |
| HFrEF_S30 | 3630 | INS |
| HFrEF_S30 | 2697 | GJA1 |
| HFrEF_S30 | 4607 | MYBPC3 |
| HFrEF_S30 | 3784 | KCNQ1 |
| HFrEF_S30 | 5728 | PTEN |
| HFrEF_S30 | 7139 | TNNT2 |
| HFrEF_S30 | 4878 | NPPA |
| HFrEF_S30 | 3586 | IL10 |
| HFrEF_S30 | 51422 | PRKAG2 |
| HFrEF_S30 | 1756 | DMD |
| HFrEF_S30 | 348 | APOE |
| HFrEF_S30 | 7137 | TNNI3 |
| HFrEF_S30 | 5428 | POLG |
| HFrEF_S30 | 6910 | TBX5 |
| HFrEF_S30 | 213 | ALB |
| HFrEF_S30 | 2627 | GATA6 |
| HFrEF_S30 | 5594 | MAPK1 |
| HFrEF_S30 | 3845 | KRAS |
| HFrEF_S30 | 675 | BRCA2 |
| HFrEF_S30 | 673 | BRAF |
| HFrEF_S30 | 6262 | RYR2 |
| HFrEF_S30 | 7040 | TGFB1 |
| HFrEF_S30 | 4846 | NOS3 |
| HFrEF_S30 | 1401 | CRP |
| HFrEF_S30 | 2260 | FGFR1 |
| HFrEF_S30 | 2200 | FBN1 |
| HFrEF_S30 | 1674 | DES |
| HFrEF_S30 | 4524 | MTHFR |
| HFrEF_S30 | 5290 | PIK3CA |
| HFrEF_S30 | 7276 | TTR |
| HFrEF_S30 | 3553 | IL1B |
| HFrEF_S30 | 2316 | FLNA |
| HFrEF_S30 | 3123 | HLA-DRB1 |
| HFrEF_S30 | 5468 | PPARG |
| HFrEF_S30 | 5972 | REN |
| HFrEF_S30 | 2261 | FGFR3 |
| HFrEF_S30 | 3717 | JAK2 |
| HFrEF_S30 | 3458 | IFNG |
| HFrEF_S30 | 3479 | IGF1 |
| HFrEF_S30 | 4204 | MECP2 |
| HFrEF_S30 | 5894 | RAF1 |
| HFrEF_S30 | 9464 | HAND2 |
| HFrEF_S30 | 25 | ABL1 |
| HFrEF_S30 | 1906 | EDN1 |
| HFrEF_S30 | 2099 | ESR1 |
| HFrEF_S30 | 1499 | CTNNB1 |
| HFrEF_S30 | 2147 | F2 |
| HFrEF_S30 | 185 | AGTR1 |
| HFrEF_S30 | 153 | ADRB1 |
| HFrEF_S30 | 5663 | PSEN1 |
| HFrEF_S30 | 70 | ACTC1 |
| HFrEF_S30 | 183 | AGT |
| HFrEF_S30 | 4838 | NODAL |
| HFrEF_S30 | 7422 | VEGFA |
| HFrEF_S30 | 57057 | TBX20 |
| HFrEF_S30 | 3757 | KCNH2 |
| HFrEF_S30 | 4763 | NF1 |
| HFrEF_S30 | 4851 | NOTCH1 |
| HFrEF_S30 | 338 | APOB |
| HFrEF_S30 | 5979 | RET |
| HFrEF_S30 | 7450 | VWF |
| HFrEF_S30 | 154 | ADRB2 |
| HFrEF_S30 | 659 | BMPR2 |
| HFrEF_S30 | 5350 | PLN |
| HFrEF_S30 | 7168 | TPM1 |
| HFrEF_S30 | 56652 | TWNK |
| HFrEF_S30 | 3952 | LEP |
| HFrEF_S30 | 6647 | SOD1 |
| HFrEF_S30 | 672 | BRCA1 |
| HFrEF_S30 | 3077 | HFE |
| HFrEF_S30 | 2318 | FLNC |
| HFrEF_S30 | 207 | AKT1 |
| HFrEF_S30 | 2022 | ENG |
| HFrEF_S30 | 3265 | HRAS |
| HFrEF_S30 | 9370 | ADIPOQ |
| HFrEF_S30 | 1080 | CFTR |
| HFrEF_S30 | 7099 | TLR4 |
| HFrEF_S30 | 3557 | IL1RN |
| HFrEF_S30 | 11155 | LDB3 |
| HFrEF_S30 | 335 | APOA1 |
| HFrEF_S30 | 627 | BDNF |
| HFrEF_S30 | 3576 | CXCL8 |
| HFrEF_S30 | 291 | SLC25A4 |
| HFrEF_S30 | 3767 | KCNJ11 |
| HFrEF_S30 | 6833 | ABCC8 |
| HFrEF_S30 | 472 | ATM |
| HFrEF_S30 | 4313 | MMP2 |
| HFrEF_S30 | 88 | ACTN2 |
| HFrEF_S30 | 2335 | FN1 |
| HFrEF_S30 | 5054 | SERPINE1 |
| HFrEF_S30 | 79147 | FKRP |
| HFrEF_S30 | 775 | CACNA1C |
| HFrEF_S30 | 2006 | ELN |
| HFrEF_S30 | 5318 | PKP2 |
| HFrEF_S30 | 1956 | EGFR |
| HFrEF_S30 | 1493 | CTLA4 |
| HFrEF_S30 | 79188 | TMEM43 |
| HFrEF_S30 | 2033 | EP300 |
| HFrEF_S30 | 4318 | MMP9 |
| HFrEF_S30 | 282996 | RBM20 |
| HFrEF_S30 | 58 | ACTA1 |
| HFrEF_S30 | 959 | CD40LG |
| HFrEF_S30 | 2717 | GLA |
| HFrEF_S30 | 2778 | GNAS |
| HFrEF_S30 | 1282 | COL4A1 |
| HFrEF_S30 | 3481 | IGF2 |
| HFrEF_S30 | 6347 | CCL2 |
| HFrEF_S30 | 1277 | COL1A1 |
| HFrEF_S30 | 50484 | RRM2B |
| HFrEF_S30 | 6389 | SDHA |
| HFrEF_S30 | 1909 | EDNRA |
| HFrEF_S30 | 3920 | LAMP2 |
| HFrEF_S30 | 948 | CD36 |
| HFrEF_S30 | 3815 | KIT |
| HFrEF_S30 | 3119 | HLA-DQB1 |
| HFrEF_S30 | 7056 | THBD |
| HFrEF_S30 | 4306 | NR3C2 |
| HFrEF_S30 | 6774 | STAT3 |
| HFrEF_S30 | 4023 | LPL |
| HFrEF_S30 | 7421 | VDR |
| HFrEF_S30 | 5311 | PKD2 |
| HFrEF_S30 | 4508 | MT-ATP6 |
| HFrEF_S30 | 50943 | FOXP3 |
| HFrEF_S30 | 695 | BTK |
| HFrEF_S30 | 859 | CAV3 |
| HFrEF_S30 | 650 | BMP2 |
| HFrEF_S30 | 7414 | VCL |
| HFrEF_S30 | 9531 | BAG3 |
| HFrEF_S30 | 6901 | TAFAZZIN |
| HFrEF_S30 | 5444 | PON1 |
| HFrEF_S30 | 2702 | GJA5 |
| HFrEF_S30 | 3690 | ITGB3 |
| HFrEF_S30 | 5167 | ENPP1 |
| HFrEF_S30 | 5373 | PMM2 |
| HFrEF_S30 | 4633 | MYL2 |
| HFrEF_S30 | 950 | SCARB2 |
| HFrEF_S30 | 4512 | MT-CO1 |
| HFrEF_S30 | 29954 | POMT2 |
| HFrEF_S30 | 4353 | MPO |
| HFrEF_S30 | 3949 | LDLR |
| HFrEF_S30 | 7132 | TNFRSF1A |
| HFrEF_S30 | 3383 | ICAM1 |
| HFrEF_S30 | 8243 | SMC1A |
| HFrEF_S30 | 2548 | GAA |
| HFrEF_S30 | 5308 | PITX2 |
| HFrEF_S30 | 3728 | JUP |
| HFrEF_S30 | 8878 | SQSTM1 |
| HFrEF_S30 | 9927 | MFN2 |
| HFrEF_S30 | 3762 | KCNJ5 |
| HFrEF_S30 | 3558 | IL2 |
| HFrEF_S30 | 4137 | MAPT |
| HFrEF_S30 | 4519 | MT-CYB |
| HFrEF_S30 | 6469 | SHH |
| HFrEF_S30 | 2153 | F5 |
| HFrEF_S30 | 5265 | SERPINA1 |
| HFrEF_S30 | 567 | B2M |
| HFrEF_S30 | 6654 | SOS1 |
| HFrEF_S30 | 4609 | MYC |
| HFrEF_S30 | 4535 | MT-ND1 |
| HFrEF_S30 | 2263 | FGFR2 |
| HFrEF_S30 | 1024 | CDK8 |
| HFrEF_S30 | 55687 | TRMU |
| HFrEF_S30 | 595 | CCND1 |
| HFrEF_S30 | 1387 | CREBBP |
| HFrEF_S30 | 3565 | IL4 |
| HFrEF_S30 | 4843 | NOS2 |
| HFrEF_S30 | 7042 | TGFB2 |
| HFrEF_S30 | 5443 | POMC |
| HFrEF_S30 | 355 | FAS |
| HFrEF_S30 | 2253 | FGF8 |
| HFrEF_S30 | 6622 | SNCA |
| HFrEF_S30 | 2056 | EPO |
| HFrEF_S30 | 84665 | MYPN |
| HFrEF_S30 | 2776 | GNAQ |
| HFrEF_S30 | 7010 | TEK |
| HFrEF_S30 | 5727 | PTCH1 |
| HFrEF_S30 | 1829 | DSG2 |
| HFrEF_S30 | 3827 | KNG1 |
| HFrEF_S30 | 8291 | DYSF |
| HFrEF_S30 | 5950 | RBP4 |
| HFrEF_S30 | 10060 | ABCC9 |
| HFrEF_S30 | 4669 | NAGLU |
| HFrEF_S30 | 4514 | MT-CO3 |
| HFrEF_S30 | 1769 | DNAH8 |
| HFrEF_S30 | 4312 | MMP1 |
| HFrEF_S30 | 1410 | CRYAB |
| HFrEF_S30 | 462 | SERPINC1 |
| HFrEF_S30 | 3778 | KCNMA1 |
| HFrEF_S30 | 4598 | MVK |
| HFrEF_S30 | 8048 | CSRP3 |
| HFrEF_S30 | 1440 | CSF3 |
| HFrEF_S30 | 6392 | SDHD |
| HFrEF_S30 | 9421 | HAND1 |
| HFrEF_S30 | 652 | BMP4 |
| HFrEF_S30 | 2629 | GBA |
| HFrEF_S30 | 2218 | FKTN |
| HFrEF_S30 | 1029 | CDKN2A |
| HFrEF_S30 | 1376 | CPT2 |
| HFrEF_S30 | 10585 | POMT1 |
| HFrEF_S30 | 825 | CAPN3 |
| HFrEF_S30 | 7043 | TGFB3 |
| HFrEF_S30 | 3162 | HMOX1 |
| HFrEF_S30 | 3091 | HIF1A |
| HFrEF_S30 | 2064 | ERBB2 |
| HFrEF_S30 | 4893 | NRAS |
| HFrEF_S30 | 836 | CASP3 |
| HFrEF_S30 | 3753 | KCNE1 |
| HFrEF_S30 | 3559 | IL2RA |
| HFrEF_S30 | 3030 | HADHA |
| HFrEF_S30 | 920 | CD4 |
| HFrEF_S30 | 8557 | TCAP |
| HFrEF_S30 | 3606 | IL18 |
| HFrEF_S30 | 6597 | SMARCA4 |
| HFrEF_S30 | 9742 | IFT140 |
| HFrEF_S30 | 51594 | NBAS |
| HFrEF_S30 | 367 | AR |
| HFrEF_S30 | 6532 | SLC6A4 |
| HFrEF_S30 | 4540 | MT-ND5 |
| HFrEF_S30 | 57190 | SELENON |
| HFrEF_S30 | 2475 | MTOR |
| HFrEF_S30 | 4314 | MMP3 |
| HFrEF_S30 | 1278 | COL1A2 |
| HFrEF_S30 | 7076 | TIMP1 |
| HFrEF_S30 | 3075 | CFH |
| HFrEF_S30 | 7018 | TF |
| HFrEF_S30 | 1565 | CYP2D6 |
| HFrEF_S30 | 11093 | ADAMTS13 |
| HFrEF_S30 | 10269 | ZMPSTE24 |
| HFrEF_S30 | 846 | CASR |
| HFrEF_S30 | 6387 | CXCL12 |
| HFrEF_S30 | 4210 | MEFV |
| HFrEF_S30 | 5617 | PRL |
| HFrEF_S30 | 1000 | CDH2 |
| HFrEF_S30 | 773 | CACNA1A |
| HFrEF_S30 | 2152 | F3 |
| HFrEF_S30 | 7170 | TPM3 |
| HFrEF_S30 | 5664 | PSEN2 |
| HFrEF_S30 | 133 | ADM |
| HFrEF_S30 | 1585 | CYP11B2 |
| HFrEF_S30 | 3908 | LAMA2 |
| HFrEF_S30 | 25974 | MMACHC |
| HFrEF_S30 | 7048 | TGFBR2 |
| HFrEF_S30 | 7097 | TLR2 |
| HFrEF_S30 | 1605 | DAG1 |
| HFrEF_S30 | 1471 | CST3 |
| HFrEF_S30 | 4541 | MT-ND6 |
| HFrEF_S30 | 64135 | IFIH1 |
| HFrEF_S30 | 3958 | LGALS3 |
| HFrEF_S30 | 6609 | SMPD1 |
| HFrEF_S30 | 54790 | TET2 |
| HFrEF_S30 | 7412 | VCAM1 |
| HFrEF_S30 | 55624 | POMGNT1 |
| HFrEF_S30 | 2875 | GPT |
| HFrEF_S30 | 1991 | ELANE |
| HFrEF_S30 | 55811 | ADCY10 |
| HFrEF_S30 | 1234 | CCR5 |
| HFrEF_S30 | 1287 | COL4A5 |
| HFrEF_S30 | 287 | ANK2 |
| HFrEF_S30 | 4534 | MTM1 |
| HFrEF_S30 | 114548 | NLRP3 |
| HFrEF_S30 | 1281 | COL3A1 |
| HFrEF_S30 | 7054 | TH |
| HFrEF_S30 | 2353 | FOS |
| HFrEF_S30 | 2010 | EMD |
| HFrEF_S30 | 7852 | CXCR4 |
| HFrEF_S30 | 5286 | PIK3C2A |
| HFrEF_S30 | 140628 | GATA5 |
| HFrEF_S30 | 9992 | KCNE2 |
| HFrEF_S30 | 11232 | POLG2 |
| HFrEF_S30 | 2170 | FABP3 |
| HFrEF_S30 | 29925 | GMPPB |
| HFrEF_S30 | 27063 | ANKRD1 |
| HFrEF_S30 | 5243 | ABCB1 |
| HFrEF_S30 | 4538 | MT-ND4 |
| HFrEF_S30 | 958 | CD40 |
| HFrEF_S30 | 8626 | TP63 |
| HFrEF_S30 | 3480 | IGF1R |
| HFrEF_S30 | 1583 | CYP11A1 |
| HFrEF_S30 | 2539 | G6PD |
| HFrEF_S30 | 6696 | SPP1 |
| HFrEF_S30 | 3596 | IL13 |
| HFrEF_S30 | 3486 | IGFBP3 |
| HFrEF_S30 | 3759 | KCNJ2 |
| HFrEF_S30 | 100 | ADA |
| HFrEF_S30 | 324 | APC |
| HFrEF_S30 | 54205 | CYCS |
| HFrEF_S30 | 4311 | MME |
| HFrEF_S30 | 488 | ATP2A2 |
| HFrEF_S30 | 596 | BCL2 |
| HFrEF_S30 | 3791 | KDR |
| HFrEF_S30 | 4221 | MEN1 |
| HFrEF_S30 | 4852 | NPY |
| HFrEF_S30 | 2070 | EYA4 |
| HFrEF_S30 | 2784 | GNB3 |
| HFrEF_S30 | 6326 | SCN2A |
| HFrEF_S30 | 841 | CASP8 |
| HFrEF_S30 | 26503 | SLC17A5 |
| HFrEF_S30 | 4151 | MB |
| HFrEF_S30 | 3356 | HTR2A |
| HFrEF_S30 | 57817 | HAMP |
| HFrEF_S30 | 4634 | MYL3 |
| HFrEF_S30 | 217 | ALDH2 |
| HFrEF_S30 | 10345 | TRDN |
| HFrEF_S30 | 6352 | CCL5 |
| HFrEF_S30 | 3240 | HP |
| HFrEF_S30 | 51738 | GHRL |
| HFrEF_S30 | 6403 | SELP |
| HFrEF_S30 | 5327 | PLAT |
| HFrEF_S30 | 1837 | DTNA |
| HFrEF_S30 | 3552 | IL1A |
| HFrEF_S30 | 7046 | TGFBR1 |
| HFrEF_S30 | 4018 | LPA |
| HFrEF_S30 | 811 | CALR |
| HFrEF_S30 | 37 | ACADVL |
| HFrEF_S30 | 5314 | PKHD1 |
| HFrEF_S30 | 10891 | PPARGC1A |
| HFrEF_S30 | 1910 | EDNRB |
| HFrEF_S30 | 10021 | HCN4 |
| HFrEF_S30 | 3691 | ITGB4 |
| HFrEF_S30 | 2247 | FGF2 |
| HFrEF_S30 | 7498 | XDH |
| HFrEF_S30 | 5500 | PPP1CB |
| HFrEF_S30 | 2592 | GALT |
| HFrEF_S30 | 4193 | MDM2 |
| HFrEF_S30 | 1586 | CYP17A1 |
| HFrEF_S30 | 6401 | SELE |
| HFrEF_S30 | 7369 | UMOD |
| HFrEF_S30 | 2395 | FXN |
| HFrEF_S30 | 4552 | MTRR |
| HFrEF_S30 | 8074 | FGF23 |
| HFrEF_S30 | 115399 | LRRC56 |
| HFrEF_S30 | 4976 | OPA1 |
| HFrEF_S30 | 6648 | SOD2 |
| HFrEF_S30 | 56729 | RETN |
| HFrEF_S30 | 632 | BGLAP |
| HFrEF_S30 | 135 | ADORA2A |
| HFrEF_S30 | 27235 | COQ2 |
| HFrEF_S30 | 1813 | DRD2 |
| HFrEF_S30 | 4049 | LTA |
| HFrEF_S30 | 847 | CAT |
| HFrEF_S30 | 10102 | TSFM |
| HFrEF_S30 | 4513 | MT-CO2 |
| HFrEF_S30 | 4724 | NDUFS4 |
| HFrEF_S30 | 387119 | CEP85L |
| HFrEF_S30 | 2688 | GH1 |
| HFrEF_S30 | 796 | CALCA |
| HFrEF_S30 | 1188 | CLCNKB |
| HFrEF_S30 | 6337 | SCNN1A |
| HFrEF_S30 | 5465 | PPARA |
| HFrEF_S30 | 2155 | F7 |
| HFrEF_S30 | 3953 | LEPR |
| HFrEF_S30 | 1026 | CDKN1A |
| HFrEF_S30 | 2212 | FCGR2A |
| HFrEF_S30 | 2908 | NR3C1 |
| HFrEF_S30 | 9499 | MYOT |
| HFrEF_S30 | 5159 | PDGFRB |
| HFrEF_S30 | 7080 | NKX2-1 |
| HFrEF_S30 | 6714 | SRC |
| HFrEF_S30 | 60 | ACTB |
| HFrEF_S30 | 2737 | GLI3 |
| HFrEF_S30 | 3930 | LBR |
| HFrEF_S30 | 654 | BMP6 |
| HFrEF_S30 | 3181 | HNRNPA2B1 |
| HFrEF_S30 | 3981 | LIG4 |
| HFrEF_S30 | 2321 | FLT1 |
| HFrEF_S30 | 3172 | HNF4A |
| HFrEF_S30 | 4792 | NFKBIA |
| HFrEF_S30 | 1545 | CYP1B1 |
| HFrEF_S30 | 4015 | LOX |
| HFrEF_S30 | 6598 | SMARCB1 |
| HFrEF_S30 | 1889 | ECE1 |
| HFrEF_S30 | 2072 | ERCC4 |
| HFrEF_S30 | 6444 | SGCD |
| HFrEF_S30 | 657 | BMPR1A |
| HFrEF_S30 | 1436 | CSF1R |
| HFrEF_S30 | 3082 | HGF |
| HFrEF_S30 | 1788 | DNMT3A |
| HFrEF_S30 | 12 | SERPINA3 |
| HFrEF_S30 | 3605 | IL17A |
| HFrEF_S30 | 779 | CACNA1S |
| HFrEF_S30 | 4982 | TNFRSF11B |
| HFrEF_S30 | 51741 | WWOX |
| HFrEF_S30 | 1908 | EDN3 |
| HFrEF_S30 | 3117 | HLA-DQA1 |
| HFrEF_S30 | 8647 | ABCB11 |
| HFrEF_S30 | 308 | ANXA5 |
| HFrEF_S30 | 1490 | CCN2 |
| HFrEF_S30 | 2597 | GAPDH |
| HFrEF_S30 | 356 | FASLG |
| HFrEF_S30 | 5660 | PSAP |
| HFrEF_S30 | 28514 | DLL1 |
| HFrEF_S30 | 4720 | NDUFS2 |
| HFrEF_S30 | 1432 | MAPK14 |
| HFrEF_S30 | 3439 | IFNA1 |
| HFrEF_S30 | 6750 | SST |
| HFrEF_S30 | 1621 | DBH |
| HFrEF_S30 | 63976 | PRDM16 |
| HFrEF_S30 | 91624 | NEXN |
| HFrEF_S30 | 3934 | LCN2 |
| HFrEF_S30 | 9254 | CACNA2D2 |
| HFrEF_S30 | 1557 | CYP2C19 |
| HFrEF_S30 | 3684 | ITGAM |
| HFrEF_S30 | 4790 | NFKB1 |
| HFrEF_S30 | 5925 | RB1 |
| HFrEF_S30 | 6584 | SLC22A5 |
| HFrEF_S30 | 255738 | PCSK9 |
| HFrEF_S30 | 2720 | GLB1 |
| HFrEF_S30 | 57104 | PNPLA2 |
| HFrEF_S30 | 2322 | FLT3 |
| HFrEF_S30 | 4537 | MT-ND3 |
| HFrEF_S30 | 546 | ATRX |
| HFrEF_S30 | 4615 | MYD88 |
| HFrEF_S30 | 6336 | SCN10A |
| HFrEF_S30 | 947 | CD34 |
| HFrEF_S30 | 7169 | TPM2 |
| HFrEF_S30 | 8600 | TNFSF11 |
| HFrEF_S30 | 3156 | HMGCR |
| HFrEF_S30 | 55215 | FANCI |
| HFrEF_S30 | 4864 | NPC1 |
| HFrEF_S30 | 3315 | HSPB1 |
| HFrEF_S30 | 8337 | H2AC18 |
| HFrEF_S30 | 8425 | LTBP4 |
| HFrEF_S30 | 3725 | JUN |
| HFrEF_S30 | 59272 | ACE2 |
| HFrEF_S30 | 1028 | CDKN1C |
| HFrEF_S30 | 3560 | IL2RB |
| HFrEF_S30 | 2944 | GSTM1 |
| HFrEF_S30 | 6338 | SCNN1B |
| HFrEF_S30 | 1437 | CSF2 |
| HFrEF_S30 | 2936 | GSR |
| HFrEF_S30 | 2625 | GATA3 |
| HFrEF_S30 | 4208 | MEF2C |
| HFrEF_S30 | 1738 | DLD |
| HFrEF_S30 | 551 | AVP |
| HFrEF_S30 | 91942 | NDUFAF2 |
| HFrEF_S30 | 3320 | HSP90AA1 |
| HFrEF_S30 | 4536 | MT-ND2 |
| HFrEF_S30 | 1824 | DSC2 |
| HFrEF_S30 | 4205 | MEF2A |
| HFrEF_S30 | 2243 | FGA |
| HFrEF_S30 | 9997 | SCO2 |
| HFrEF_S30 | 142 | PARP1 |
| HFrEF_S30 | 5175 | PECAM1 |
| HFrEF_S30 | 4179 | CD46 |
| HFrEF_S30 | 54539 | NDUFB11 |
| HFrEF_S30 | 3032 | HADHB |
| HFrEF_S30 | 7840 | ALMS1 |

Table S3. Hyperuricemia genes with score value larger than 5.

| Gene Symbol | Disease | Gene Entrez ID |
| --- | --- | --- |
| ALDH16A1 | Hyperuricemia | 126133 |
| SLC2A9 | Hyperuricemia | 56606 |
| HPRT1 | Hyperuricemia | 3251 |
| ABCG2 | Hyperuricemia | 9429 |
| ADRB3 | Hyperuricemia | 155 |
| ALDH2 | Hyperuricemia | 217 |
| APOA1 | Hyperuricemia | 335 |
| APOC3 | Hyperuricemia | 345 |
| APOE | Hyperuricemia | 348 |
| ESR1 | Hyperuricemia | 2099 |
| GCKR | Hyperuricemia | 2646 |
| HLA-B | Hyperuricemia | 3106 |
| IL10 | Hyperuricemia | 3586 |
| KCNQ1 | Hyperuricemia | 3784 |
| LRP2 | Hyperuricemia | 4036 |
| MTHFR | Hyperuricemia | 4524 |
| PDZK1 | Hyperuricemia | 5174 |
| SLC17A1 | Hyperuricemia | 6568 |
| TNF | Hyperuricemia | 7124 |
| UMOD | Hyperuricemia | 7369 |
| SLC17A3 | Hyperuricemia | 10786 |
| LRRC16A | Hyperuricemia | 55604 |
| SLC22A11 | Hyperuricemia | 55867 |
| SLC22A12 | Hyperuricemia | 116085 |
| SARS2 | Hyperuricemia | 54938 |
| REN | Hyperuricemia | 5972 |
| SLC37A4 | Hyperuricemia | 2542 |
| G6PC1 | Hyperuricemia | 2538 |
| HNF1B | Hyperuricemia | 6928 |
| PRPS1 | Hyperuricemia | 5631 |
| PFKM | Hyperuricemia | 5213 |
| MUC1 | Hyperuricemia | 4582 |
| XDH | Hyperuricemia | 7498 |
| GAA | Hyperuricemia | 2548 |
| INS | Hyperuricemia | 3630 |
| SEC61A1 | Hyperuricemia | 29927 |
| PPARG | Hyperuricemia | 5468 |
| GPATCH8 | Hyperuricemia | 23131 |
| GPT | Hyperuricemia | 2875 |
| CRP | Hyperuricemia | 1401 |
| GGT1 | Hyperuricemia | 2678 |
| ALB | Hyperuricemia | 213 |
| LEP | Hyperuricemia | 3952 |
| NLRP3 | Hyperuricemia | 114548 |

Table S4. Baseline characteristics of participants from the NIS database.

| **Characteristic** | Patients without HFrEF (n=17302475) | Patients with HFrEF (n=866962) | *P* Value |
| --- | --- | --- | --- |
| Age, yrs (mean±SD) | 57.29±20.362 | 69.49±14.164 | ＜0.001 |
| Female sex, % | 10173701 (58.8) | 319337 (36.8) | ＜0.001 |
| **Race** |  |  | ＜0.001 |
| White, % | 11254744 (67.4) | 563033 (66.9) |  |
| African American, % | 2494170 (14.9) | 164972 (19.6) |  |
| Hispanic, % | 1886689 (11.3) | 69663 (8.3) |  |
| Asian/Pacific Islander, % | 464179 (2.8) | 17575 (2.1) |  |
| Native American, % | 105670 (0.6) | 4757 (0.6) |  |
| Other races, % | 505177 (3.0) | 21948 (2.6) |  |
| **Comorbidity** |  |  |  |
| Hyperuricemia, % | 12166 (0.07) | 1302 (0.2) | ＜0.001 |
| Coagulopathy, % | 311227 (1.8) | 25533 (2.9) | ＜0.001 |
| Obesity, % | 2641674 (15.3) | 150095 (17.3) | ＜0.001 |
| Fluid and electrolyte disorders, % | 4004711 (23.1) | 317737 (36.6) | ＜0.001 |
| Hyperlipemia, % | 5420774 (31.3) | 448995 (51.8) | ＜0.001 |
| Hypothyroidism, % | 6759 (0.04) | 578 (0.066) | ＜0.001 |
| Valvular heart disease, % | 617321 (3.6) | 117078 (13.5) | ＜0.001 |
| Chronic kidney disease, % | 2614036 (15.1) | 377034 (43.5) | ＜0.001 |
| Chronic liver disease, % | 949721 (5.5) | 63179 (7.3) | ＜0.001 |
| Chronic lung disease, % | 3792215 (21.9) | 306363 (35.3) | ＜0.001 |
| Diabetes mellitus, % | 4450913 (25.7) | 391248 (45.1) | ＜0.001 |
| Chronic anemia, % | 835423 (4.8) | 61044 (7.0) | ＜0.001 |
| Carotid artery disease, % | 216615 (1.3) | 16222 (1.9) | ＜0.001 |
| Peripheral vascular disease, % | 263653 (1.5) | 24717 (2.9) | ＜0.001 |
| Long-term use of oral hypoglycemic drugs, % | 702263 (4.1) | 48532 (5.6) | ＜0.001 |
| **Previous medical history** |  |  |  |
| Prior MI, % | 825131 (4.8) | 167221 (19.5) | ＜0.001 |
| Prior PCI, % | 848095 (4.9) | 136338 (15.7) | ＜0.001 |
| Prior CABG, % | 1323290 (7.6) | 233565 (26.9) | ＜0.001 |
| Prior stroke, % | 359212 (2.1) | 25775 (3.0) | ＜0.001 |
| Prior cardiac pacemaker, % | 409129 (2.4) | 66824 (7.7) | ＜0.001 |
| Prior LVAD, % | 3659 (0.02) | 6161 (0.7) | ＜0.001 |
| Alcohol use, % | 1037779 (6.0) | 40698 (4.7) | ＜0.001 |
| Tobacco abuse, % | 3443760 (19.9) | 249749 (28.8) | ＜0.001 |
| In-hospital mortality, % | 359870 (2.1) | 44141 (5.1) | ＜0.001 |
| Length of hospital stay, days | 4.66±6.267 | 6.37±7.527 | ＜0.001 |
| Total charges | 52283.83±85146.039 | 79313.65±141142.929 | ＜0.001 |

CABG indicates coronary artery bypass grafting; LVAD, left ventricular assist device; PCI, percutaneous coronary intervention; MI, myocardial infarction; ECHO, extracorporeal membrane oxygenation.

Table S5. Baseline characteristics of the HFrEF patients in Xianyang cohort study.

| Characteristics | Total  (n=102) | UA<420 μmmol/L  (n=86) | UA≥420 μmmol/L  (n=16) | *P* value |
| --- | --- | --- | --- | --- |
| **Demographics** |  |  |  |  |
| Age, years | 61.13±9.14 | 61.36±9.11 | 59.88±9.49 | 0.83 |
| Male, % | 63 (61.7) | 50 (58.1) | 13 (81.3) | 0.08 |
| BMI, kg/m^2^ | 23.32±3.37 | 23.20±3.23 | 23.94±4.09 | 0.41 |
| **Etiology, %** |  |  |  | 0.152 |
| Dilated cardiomyopathy | 60 (58.8) | 48 (55.8) | 12 (75) |  |
| other | 42 (41.2) | 38 (44.2) | 4 (59.4) |  |
| **Medical history, %** |  |  |  |  |
| Hypertension | 33 (32.7) | 32 (37.6) | 1(6.3) | 0.014 |
| Diabetes mellitus | 11 (10.8) | 8 (9.3) | 3 (18.8) | 0.263 |
| CAD | 36 (35.3) | 31 (36.0) | 5 (31.3) | 0.712 |
| Smoking | 54 (52.9) | 45 (52.3) | 9 (56.3) | 0.773 |
| **Laboratory data** |  |  |  |  |
| Scr, umol/L | 78.56±15.51 | 77.13±15.71 | 86.25±12.07 | 0.12 |
| BUN mmol/L | 6.97±2.11 | 6.71±2.00 | 8.36±2.18 | 0.51 |
| UA, ummol/L | 330.08±89.62 | 300.91±61.29 | 486.88±41.12 | 0.06 |
| eGFR, ml/min/1.73 m^2^ | 71.70±20.98 | 71.89±21.65 | 70.67±17.50 | 0.401 |
| NT-proBNP, ng/L | 2563.47±3036.51 | 2176.68±2587.04 | 4594.10±4311.02 | 0.01 |
| QRS, ms | 126.26±25.74 | 126.30±25.75 | 126.07±26.65 | 0.99 |
| **Echocardiographic data** |  |  |  |  |
| LV EDD, mm | 71.44±7.92 | 71.27±8.18 | 72.38±6.44 | 0.072 |
| LV ESD, mm | 59.75±7.85 | 59.45±8.18 | 61.31±5.69 | 0.092 |
| LVEF, % | 31.56±7.85 | 31.81±5.64 | 30.19±5.59 | 0.994 |
| **Follow-up time, months** | 33.44±8.67 | 33.90±8.57 | 31.00±9.15 | 0.993 |
| **Medication use, %** |  |  |  |  |
| ACE inhibitor | 89 (87.3) | 76 (88.4) | 13 (81.3) | 0.433 |
| ARB | 6 (5.8) | 4 (4.7) | 2 (12.5) | 0.221 |
| β-blocker | 91 (89.2) | 77 (89.5) | 14 (87.5) | 0.810 |
| Aldosterone receptor antagonist | 80 (78.4) | 67 (77.9) | 13 (81.3) | 0.765 |
| **NYHA, %** |  |  |  | 0.673 |
| I | 17 (16.8) | 13 (15.3) | 4 (25.0) |  |
| II | 58 (57.4) | 50 (58.8) | 8 (50) |  |
| III | 23 (22.8) | 19 (22.4) | 4 (25) |  |
| IV | 3 (3.0) | 3 (3.5) | 0 (0) |  |
| All-cause mortality | 26 (25.5) | 19 (22.1) | 7 (43.8) | 0.068 |
| Rehospitalization | 59 (60.2) | 46 (56.1) | 13 (81.3) | 0.060 |
| Composite outcomes events | 70 (68.6) | 56 (65.1) | 14 (87.5) | 0.076 |

BMI, body mass index; CAD, coronary artery disease; SBP, systolic blood pressure; DBP, diastolic blood pressure; Scr, serum creatinine; BUN, blood urea nitrogen; UA, Uric Acid; eGFR, estimated glomerular filtration rate; NT-pro BNP, N-terminal B-type natriuretic peptide; LVEDD, left ventricular end-diastolic diameter; LVESD, left ventricular end-systolic diameter; LVEF, left ventricular ejection fraction; ACE, angiotensin converting enzyme; ARB, angiotensin receptor blocker. Values are percent or means ± SD.
