# Supplementary material for: Uric Acid Predicts Recovery of Left Ventricular Function and Adverse Events in Heart Failure With Reduced Ejection Fraction: Potential Mechanistic Insight From Network Analyses
Source: Front Cardiovasc Med. 2022 Jul 15;9:853870. doi: 10.3389/fcvm.2022.853870 (PMC9334530; doi:10.3389/fcvm.2022.853870)
Supplement: Supplementary file 2 [file Data_Sheet_2.docx]

**Materials and methods**

**The National Inpatient Sample (NIS) database analysis**

*Study population from the NIS database*

The first part of the present study was conducted using data for the period from 2016 to 2018 from the NIS database, the largest inpatient, publicly available database in the USA, which was developed by the Agency for Healthcare Research and Quality.

The NIS database was sampled between 2016 and 2018 to identify hospitalizations in which patients had a primary diagnosis of heart failure with reduced ejection fraction (HFrEF) with hyperuricemia or without hyperuricemia, specified by International Classification of Diseases, Tenth Revision, Clinical Modification (ICD-10-CM) codes. A total of 8659014 hospitalizations were identified (Figure S1). Patients aged<18 years or with missing data were excluded from the study.

We also collected additional information on patient demographic characteristics, cardiovascular risk profile including history of coronary artery disease (CAD), smoking status, dyslipidemia, chronic hypertension, prior acute MI or history of coronary artery bypass graft (CABG), and previous stroke or transient ischemic attack, acute organ failure, mechanical circulatory support using ICD-10-CM codes and procedural codes (Table S1).

*Study Outcomes*

The outcomes of the study were the trends of (1) burden of comorbidities, (2) inpatient mortality, (3) mean length of stay, and (4) mean cost of stay.

**Xianyang Chronic Heart Failure Prospective Cohort Study**

***Study Population***

The second part of this study was proceeded within the Xianyang Chronic Heart Failure Prospective Cohort, an ongoing prospective cohort study focusing on preventing the progression of chronic heart failure in Western-China rural areas of Shaanxi province. The study began in 2014 and the individuals were enrolled from two towns (Xunyi, and Jingyang) in Xianyang, Shaanxi, China. All of these included patients regularly attended the heart failure outpatients’ clinic in Xunyi and Jingyang hospitals.

There were 102 HFrEF patients were enrolled and identified 57 heart failure with recovery ejection fraction (HFrecEF) patients, defined as a reduced left ventricle ejection fraction (LVEF) <40% (baseline echocardiogram), whose LVEF had increased by at least 10% or normalized (>40%) on evidence-based medical therapies for 1 year after follow-up echocardiogram[1-3] (Figure S1). Baseline characteristics including demographic characteristics, medication, medical history, drinking history, and cigarette smoking history were acquired through a questionnaire. Height, body weight, and blood pressure were measured by trained and qualified personnel. Body mass index (BMI) was computed as weight in kilograms divided by the square of the height.

Patients were followed up in detail at baseline and a minimum of 6 months. Follow-up was done by telephone to the patients’ homes or gave the calls to their physicians, regular outpatient follow-up, or electronic hospital records to follow up and determine the survival of the patients. In order to analysis, patients alive at this time were censored as alive at the time of the last contact. The primary endpoints were all-cause death, or hospitalized for severe HF. Composite events were defined as hospitalized for severe HF, all-cause death or both hospitalized for severe HF and all-cause death.

***Inclusion and exclusion criteria***

The inclusion criteria were as follows: aged 18 to 80 years; newly diagnosed outpatients, hospitalized patients with typical symptoms and signs of heart failure (HF) or diagnosed HF patients; echocardiography showed LVEF<40%; echocardiographic evidence of left ventricular diastolic dysfunction; All patients were included after stabilization of both clinical status and medication. Exclusion criteria were HF related to congenital heart diseases, vascular disease or pericardial disease; acute HF, chronic obstructive pulmonary disease, serious blood disease, autoimmune disease, active infection or malignancy; Women with pregnancy and lactation, dementia or mental disorder, patients are not suitable for this research based on researchers' evaluation. We also excluded the patients with a diagnosis of gout, or severe kidney disease with a renal replacement therapy (dialysis or ultrafiltration). Furthermore, we excluded patients treated with Uric Acid (UA)-lowering drugs to avoid another confounding factor. This study was authorized by the ethics committee of the First Affiliated Hospital of Xi’an Jiaotong University and was performed conform to the declaration of the Helsinki Declaration. All patients signed the informed consent forms.

***Biochemical Parameter and Echocardiography Measurements***

Fasting venous blood samples were collected by experienced nurses in the morning after fasting for 8-10 hours. Venous blood samples were immediately centrifuged at 3000g for 10 minutes and stored at -80°C until analysis. The patients’ first urine in the morning were obtained and were kept frozen at -40°C until analysis. Some biomarkers, including hemoglobin, serum creatinine, blood urea nitrogen, urinary uric acid, N-terminal B-type natriuretic peptide (NT-proBNP), were measured by an automatic biochemical analyzer. Echocardiography was performed by experienced echocardiologists at baseline and first year follow-up, LVEF, LV end-diastolic diameter (LVEDD), LV end-systolic diameter (LVESD), LV end-diastolic volume (LVEDV), LV end- systolic volume (LVESV), LV fractional shortening (LVFS) were obtained. Hyperuricemia was classified as serum UA≥420 μmmol/L in men, and UA≥360μmmol/L in women.

***Statistical analysis***

Propensity score matching (PSM), a method to balance covariates in two groups by reducing the selection bias, was conducted to match HFrEF patients with hyperuricemia to patients without hyperuricemia. In our study, we included variables that may be associated with the outcome of HFrEF patients of NIS database in the propensity score model. Matching factors for PSM including age, sex, race, hypertension, diabetes, alcohol abuse, hyperlipemia, obesity, chronic kidney diseases, hypothyroidism, chronic respiratory disease, cerebral infarction, coagulopathy.

Continuous variables were denoted as mean± SD and were contrasted using Student t test or Mann-Whitney t test as appropriate. Categorical data were expressed as numbers and percentages and were compared using Pearson’s chi-square test (χ^2^) and Fisher’s exact test. Outcomes were compared between the HFrEF with hyperuricemia and without hyperuricemia groups. Factors associated with the risk of the HFrEF were examined with univariate and multivariate logistic regression.

The correlation between the LVEF and UA on the baseline visit, as well as the correlation between UA on the baseline and the LVEF on the follow-up, were assessed by the Pearson correlation coefficient. Predictive values of the baseline UA for recovery of LVEF and prognosis were tested with receiver operating characteristic curves (ROC), quantified by calculating the area under the ROC curve (AUC) and the 95% confidence interval (CI). The association between UA on the baseline visit and the LVEF on the follow-up visit were examined via logistic regression model. Multivariate Cox of regression analysis was performed to evaluate the independent association between hyperuricemia and the outcome events of the HFrEF patients. We first performed univariate cox regression analysis, the variables of age, female, BMI, Smoking, hypertension, diabetes mellitus, coronary heart disease, UA, baseline LVEF, NT-proBNP, heart rate, urea nitrogen and eGRF which with *P*<0.1 were included in multivariate cox regression analysis. Cumulative survival curve for endpoints was constructed using the Kaplan-Meier method and the log-rank test was used to find the difference. *P* value <0.05 was considered statistically significant. Statistical analyses were performed using SPSS version 25 (IBM, Armonk, NY) and R version 3.5 (version 3.6.3, R Core Team).

***Network analysis***

*Manual Curation of disease genes*

Specifically, we first collected HFrEF genes via searching the terms “Heart failure with a reduced ejection fraction” in GeneCards (<https://www.genecards.org/>) database and reserved those HFrEF genes with score value higher than 30 (Table S2). We next classified these HFrEF genes into four types of datasets according to their score values: 1) Score > 30 (HFrEF_S30); 2) Score > 40 (HFrEF_S40); 3) Score > 50 (HFrEF_S50); 4) Score > 60 (HFrEF_S60). Meanwhile, the Hyperuricemia genes were acquired from GeneCards database and DisGeNET (S>0.3). Only Hyperuricemia genes with score value larger than 5 were utilized in our study. After removing the duplicates, 44 Hyperuricemia genes were finally obtained (Table S3).

***Network analysis-based statistical models***

*Network proximity*

We define A as the set of Hyperuricemia genes and B as the set of HFrEF genes. The nearest distance between A and B ($d_{AB}$) represents the average nearest route distance of all the disease genes in Hyperuricemia gene set (A) to HFrEF gene set (B) within the human protein interactome, which can be depicted as below:

$$\left\langle d_{AB} \right\rangle=\frac{1}{\left| \left| A \right| \right|+\left\| B \right\|}\left( \sum_{a\in A} {min}_{b\in B}d\left( a,b \right)+\sum_{b\in B} {min}_{a\in A}d\left( a,b \right) \right)$$

where $d\left( a,b \right)$ represents the nearest route distance between Hyperuricemia gene $a$ and HFrEF gene$b$.

To measure the network length between gene set A and B, we randomly extracted two gene datasets from human protein interactome which have the same size and degree distribution as original gene sets. After repeating this process for 10000 times, Z-score ($z_{d}$) was computed as follows:

$$z_{d}=\frac{d-\bar{d}}{\sigma_{d}}$$

where $d$ and $\bar{d}$ were $d\left( a,b \right)$and the mean, $\sigma_{d}$ represents standard deviation of the reference distribution.

We further calculated *P* values during the permutation test. The Hyperuricemia-HFrEF gene pairs were regarded as significantly proximal if they satisfied the condition (*P* < 0.05).

***Gene overlap analysis***

In this study, we performed gene overlap analysis on different datasets to identify the common disease genes. We next applied the Fisher’s exact test to evaluate the statistical significance between hyperuricemia gene sets and HFrEF ones[4]. The *P*-values were calculated, which were subsequently corrected by Benjamini–Hochberg method. We assumed that a hyperuricemia-HFrEF gene pair was significant if its cutoff adjusted *P*-value threshold (*q*) was lower than 0.05.

1. Bozkurt B, Coats AJS, Tsutsui H, Abdelhamid CM, Adamopoulos S, Albert N, Anker SD, Atherton J, Böhm M, Butler J *et al*: **Universal definition and classification of heart failure: a report of the Heart Failure Society of America, Heart Failure Association of the European Society of Cardiology, Japanese Heart Failure Society and Writing Committee of the Universal Definition of Heart Failure: Endorsed by the Canadian Heart Failure Society, Heart Failure Association of India, Cardiac Society of Australia and New Zealand, and Chinese Heart Failure Association**. *Eur J Heart Fail* 2021, **23**(3):352-380.

2. Bozkurt B, Coats AJ, Tsutsui H, Abdelhamid M, Adamopoulos S, Albert N, Anker SD, Atherton J, Böhm M, Butler J *et al*: **Universal Definition and Classification of Heart Failure: A Report of the Heart Failure Society of America, Heart Failure Association of the European Society of Cardiology, Japanese Heart Failure Society and Writing Committee of the Universal Definition of Heart Failure**. *J Card Fail* 2021.

3. Wilcox JE, Fang JC, Margulies KB, Mann DL: **Heart Failure With Recovered Left Ventricular Ejection Fraction: JACC Scientific Expert Panel**. *J Am Coll Cardiol* 2020, **76**(6):719-734.

4. Cai C, Wu Q, Hong H, He L, Liu Z, Gu Y, Zhang S, Wang Q, Fan X, Fang J: **In silico identification of natural products from Traditional Chinese Medicine for cancer immunotherapy**. *Scientific reports* 2021, **11**(1):3332.
