# Supplementary figures and images for: Uric Acid Predicts Recovery of Left Ventricular Function and Adverse Events in Heart Failure With Reduced Ejection Fraction: Potential Mechanistic Insight From Network Analyses
Source: Front Cardiovasc Med. 2022 Jul 15;9:853870. doi: 10.3389/fcvm.2022.853870 (PMC9334530; doi:10.3389/fcvm.2022.853870)

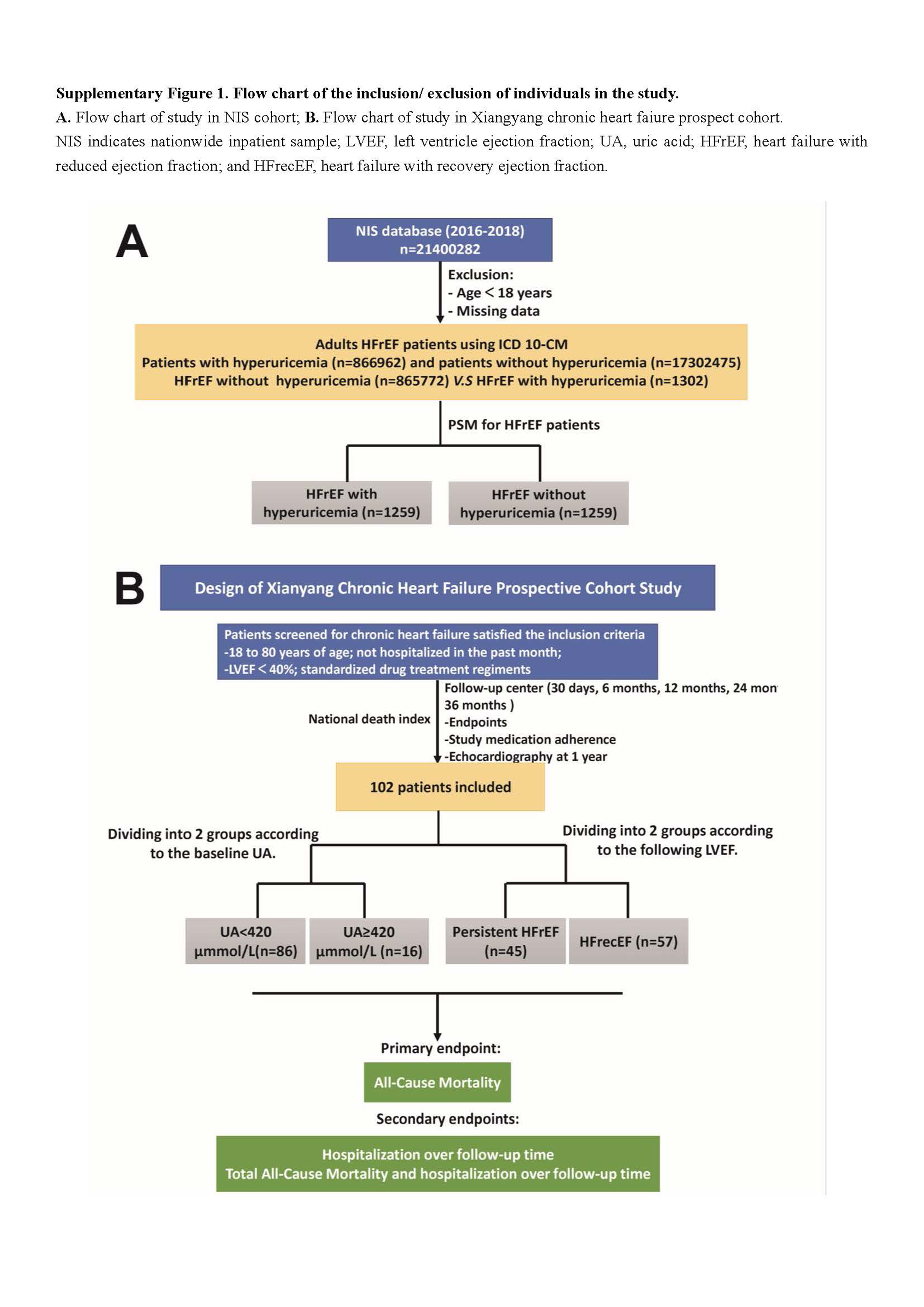

Supplement: Supplementary file 3 [file Image_1.jpg]

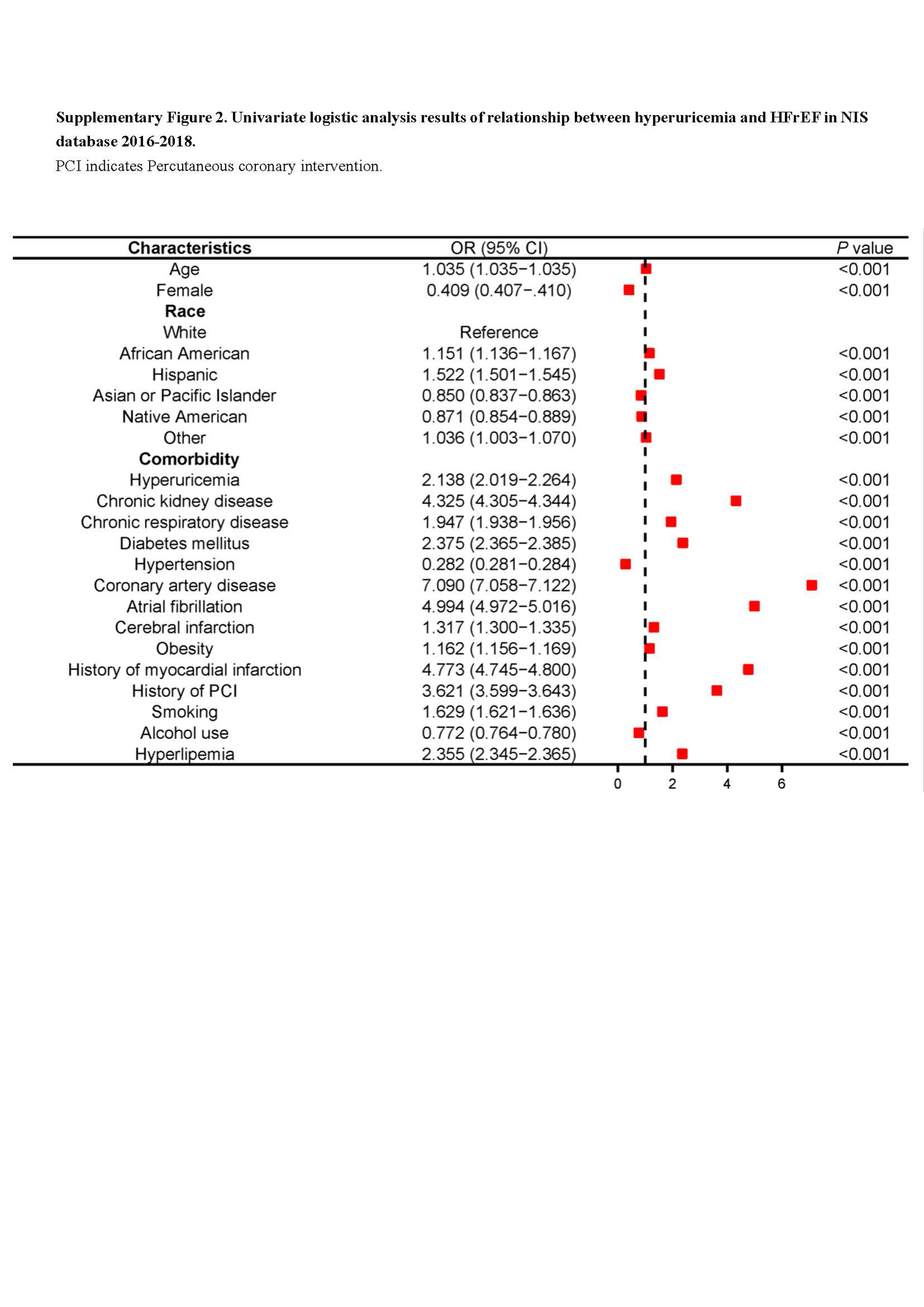

Supplement: Supplementary file 4 [file Image_2.jpg]

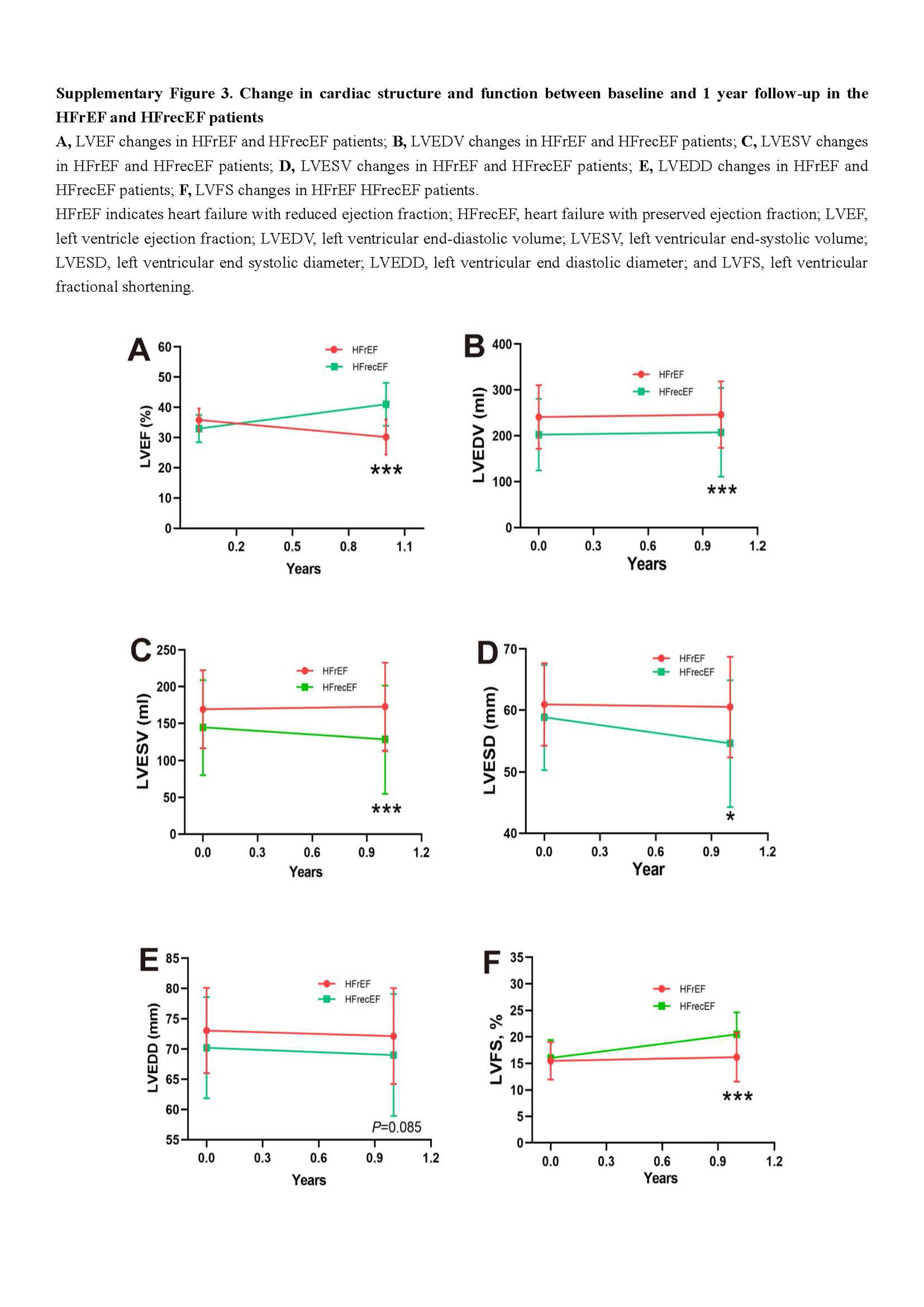

Supplement: Supplementary file 5 [file Image_3.jpg]
